# Selective inhibition of cancer cell self-renewal through a Quisinostat-histone H1.0 axis

**DOI:** 10.1038/s41467-020-15615-z

**Published:** 2020-04-14

**Authors:** Cristina Morales Torres, Mary Y. Wu, Sebastijan Hobor, Elanor N. Wainwright, Matthew J. Martin, Harshil Patel, William Grey, Eva Grönroos, Steven Howell, Joana Carvalho, Ambrosius P. Snijders, Michael Bustin, Dominique Bonnet, Paul D. Smith, Charles Swanton, Michael Howell, Paola Scaffidi

**Affiliations:** 1 Cancer Epigenetics Laboratory, Francis Crick Institute, London, NW1 1AT UK; 2 High-Throughput Screening, Francis Crick Institute, London, NW1 1AT UK; 3 Cancer Evolution and Genome Instability Laboratory, Francis Crick Institute, London, NW1 1AT UK; 4 Oncology R&D, AstraZeneca, Cambridge, CB2 0RE UK; 5 Bioinformatics and Biostatistics, Francis Crick Institute, London, NW1 1AT UK; 6 Haematopoietic Stem Cell Laboratory, Francis Crick Institute, London, NW1 1AT UK; 7 Proteomics, Francis Crick Institute, London, NW1 1AT UK; 8 Experimental Histopathology, Francis Crick Institute, London, NW1 1AT UK; 9 Center for Cancer Research, National Cancer Institute, National Institutes of Health, Bethesda, MD 20814 USA; 10 Cancer Research UK Lung Cancer Centre of Excellence, UCL Cancer Institute, University College London, London, WC1E 6BT UK; 11 UCL Cancer Institute, University College London, London, WC1E 6DD UK

**Keywords:** Cancer therapy, Tumour heterogeneity

## Abstract

Continuous cancer growth is driven by subsets of self-renewing malignant cells. Targeting of uncontrolled self-renewal through inhibition of stem cell-related signaling pathways has proven challenging. Here, we show that cancer cells can be selectively deprived of self-renewal ability by interfering with their epigenetic state. Re-expression of histone H1.0, a tumor-suppressive factor that inhibits cancer cell self-renewal in many cancer types, can be broadly induced by the clinically well-tolerated compound Quisinostat. Through H1.0, Quisinostat inhibits cancer cell self-renewal and halts tumor maintenance without affecting normal stem cell function. Quisinostat also hinders expansion of cells surviving targeted therapy, independently of the cancer types and the resistance mechanism, and inhibits disease relapse in mouse models of lung cancer. Our results identify H1.0 as a major mediator of Quisinostat’s antitumor effect and suggest that sequential administration of targeted therapy and Quisinostat may be a broadly applicable strategy to induce a prolonged response in patients.

## Abstract (web summary)

Targeting self-renewing cancer cells without deleterious side effects in normal tissues has proven challenging. Here, the authors show that the well-tolerated HDAC inhibitor Quisinostat safely inhibits tumor maintenance and disease relapse by restoring high levels of histone H1.0.

## Introduction

Acquisition of uncontrolled self-renewal ability, i.e. the ability to proliferate indefinitely in the absence of physiological stimuli, is essential for tumorigenesis^1^. However, cancer cells may lose self-renewal ability during tumor growth because of genetic or non-genetic mechanisms that act after tumor initiation^2–5^. For instance, due to genomic instability, cancer cells may accumulate deleterious secondary mutations or suffer complex genomic rearrangements that impair their fitness^4,6^. Furthermore, cell-to-cell interactions with the tumor microenvironment can alter the proliferative potential of cancer cells by inducing their differentiation^3,5,7–9^. In many cancers, only cells exhibiting primitive, undifferentiated phenotypes have long-term proliferative potential and are able to propagate the disease when transplanted into immunocompromised mice^5,8–10^. Thus, the differentiation process that naturally occurs during cancer growth inhibits self-renewal of tumor cells and effectively deprives them of malignant properties. Only cells that retain unlimited proliferative potential can fuel the long-term cancer growth and regenerate the tumor if they survive treatment. The abundance of self-renewing cells within a cancer correlates with disease aggressiveness and is predictive of patient outcome in various cancers^11–13^. Considerable effort has been made to target self-renewing cells by inhibiting stem cell-related signaling pathways, such as Wnt, Notch and Hedgehog^14,15^. However, as normal tissue homeostasis is critically dependent on these pathways, available compounds also interfere with normal stem cell function and are associated with major toxicity^14,15^. Furthermore, cross talk between signaling pathways limits the efficacy of the approach, as inhibition of a pathway is often associated with compensatory activation of an interconnected one, leading to therapeutic escape^14,15^.

The balance between cell self-renewal and differentiation is ultimately regulated by epigenetic mechanisms involving changes in chromatin and DNA methylation patterns. The linker histone H1.0, an integral component of chromatin broadly expressed in adult tissues, is a potent inhibitor of cancer cell self-renewal, which is reversibly silenced in many cancer types^16^. Within individual tumors, cells that stably repress H1.0 preserve a chromatin configuration compatible with uncontrolled self-renewal, whereas cells re-expressing H1.0 during tumor growth acquire an epigenetic state that restricts their proliferative potential^16^. Thus, variable H1.0 levels within tumors determine which cells can drive the long-term cancer growth. H1.0 represses self-renewal-related transcriptional programs by regulating nucleosome occupancy in AT-rich domains of the genome, where numerous genes that sustain uncontrolled proliferation are located^16^. Using genetic approaches, we have previously shown that restoring high H1.0 levels homogeneously across tumor cells is an effective means to impair tumor maintenance^16^. We therefore searched for chemical compounds that induce re-expression of H1.0 in cancer cells (Supplementary Fig. 1a). Here, we show that multiple HDAC inhibitors (HDACi) restore high levels of H1.0 in a large panel of cell lines from numerous cancer types and in patient-derived xenografts (PDXs). We demonstrate that Quisinostat, a potent second-generation HDACi^17^, inhibits cancer cell self-renewal, effectively halting disease maintenance and relapse. We also show that the anti-tumor effect of Quisinostat is primarily mediated by H1.0, as cells unable to re-express H1.0 are insensitive to the drug. Importantly, Quisinostat does not impair self-renewal of normal stem cells, a finding in line with the good safety profile of Quisinostat^18,19^, and HDAC inhibitors in general^20^, in patients. Identification of a well-tolerated compound as a specific inhibitor of cancer cell self-renewal and characterization of its mechanism of action provide a means to induce a durable response in patients without causing severe side effects.

## Results

### Pharmacological induction of histone H1.0

**Fig. 1:**
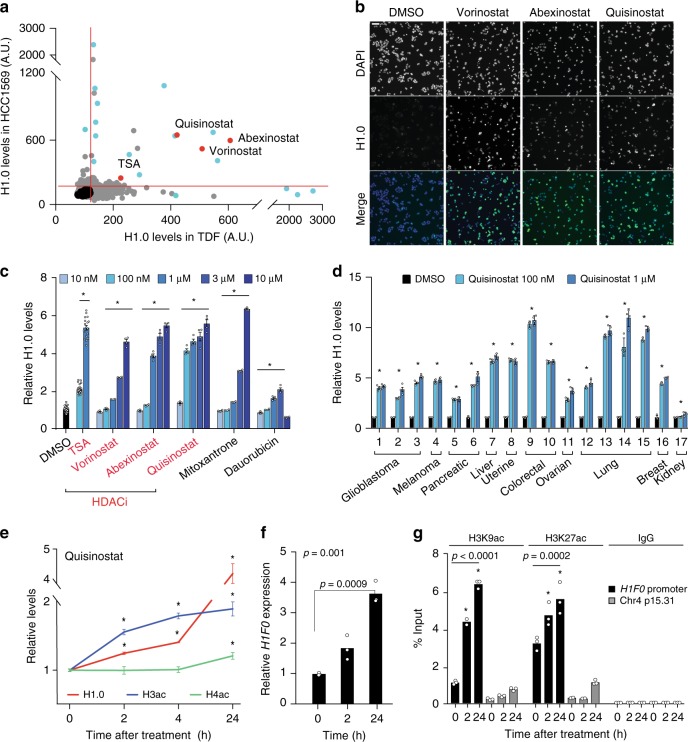
HDAC inhibitors restore high levels of H1.0 in a large panel of cancer cell lines. **a** Immunofluorescence microscopy quantifying H1.0 levels in the screen. Each dot represents the median value for each compound (*n* = 3 biological replicates). Black: DMSO, blue: compounds tested for validation, red: HDACi. **b** Representative images from one of three HCC1569 biological replicates from the screen. Scale bar: 50 µm. **c** Validation experiments in HCC1569 cells. Values represent mean ± s.e.m. from four biological replicates for each condition, except DMSO (*n* = 48), 100 nM TSA (*n* = 24) and 1 μM TSA (*n* = 18). *p-value < 0.0001 (one-way ANOVA) for each drug titration compared to DMSO. **d** Immunofluorescence microscopy quantifying H1.0 levels 24 h after Quisinostat treatment in cell lines from the indicated cancer types (cell line names listed in Supplementary Table 1). Values represent mean ± s.e.m. from four biological replicates. *p-value < 0.0001 (one-way ANOVA) comparing DMSO- and Quisinostat-treated cells. **e** Immunofluorescence microscopy quantifying levels of H1.0, acetylated H3 (H3ac) and acetylated H4 (H4ac) at the indicated times after 100 nM Quisinostat treatment of HCC1569 cells. Values represent mean ± s.e.m. from eight (H1.0) or four (H3, H4) biological replicates. *p-value < 0.01, compared to 0 h (one-tailed Student’s *t*-test). Exact *p*-values in Source Data file. **f** qRT-PCR quantifying *H1F0* expression levels in HCC1569 cells at the indicated time after treatment with 100 nM Quisinostat. Values are mean from three technical replicates. *p*-values: one-way ANOVA comparing untreated and treated (value on top) followed by Dunnett’s test (value above line). **g** ChIP-qPCR analysis of the acetylation status of the *H1F0* promoter and of a control region at the indicated times after 100 nM Quisinostat treatment. Values are mean from three technical replicates. Data are shown as relative to 1% of input. The significance of the differences between treated and untreated cells is indicated for each antibody for the *H1F0* promoter samples (one-way ANOVA, followed by Dunnett’s test). *p < 0.001. Exact *p*-values in Source Data file. Data for all graphs in Source Data file.

Unlike replication-dependent H1 subtypes, H1.0 levels respond to a variety of intrinsic and extrinsic cues, suggesting that its expression can be modulated^21^. Furthermore, although H1.0 is downregulated in self-renewing cells within tumors, high H1.0 levels are spontaneously restored in the tumor bulk, indicating that cancer cells are competent for H1.0 re-expression^16^. Focusing on annotated and characterized compounds, we searched for molecules that reversed H1.0 silencing in cancer cells (Supplementary Fig. 1a). To identify molecules of broad utility, we initially selected two human cell lines with distinct characteristics: HCC1569 breast cancer cells^22^, a slow-growing cell line of epithelial origin, and TDF transformed dermal fibroblasts^16^, a fast-growing cell line of mesenchymal origin. By screening a library of over 4000 molecules by quantitative immunofluorescence microscopy, we identified 133 compounds that induced upregulation of H1.0 after 24 h in both cell lines, with 21 molecules showing particularly high activity (Fig. 1a, b; Supplementary Fig. 1b and Supplementary Datasets 1 and 2). In addition, 12 compounds showed high activity in only one cell line (Supplementary Dataset 2). Validation experiments testing 18 primary hits confirmed a strong and dose-dependent activity for 6 compounds in HCC1569 and TDF cells and in an additional breast cancer cell line (HCC1954^22^), with levels of H1.0 increasing up to 10-fold (Fig. 1c and Supplementary Fig. 1c). Twelve other compounds showed only weak or no activity (Supplementary Fig. 1d, e and Supplementary Dataset 2). Notably, 4 of the 6 validated molecules were related, as they all belong to the HDAC inhibitor class of compounds^20^: Trichostatin A (TSA)^23^, suberanilohydroxamic acid (SAHA)/Vorinostat^24^, and the second-generation inhibitors PCI-24781/Abexinostat^25^ and JNJ-26481585/Quisinostat^17^ (Fig. 1a–c). Although characterized by distinct chemical structure, potency and stability, all molecules inhibit common targets (Supplementary Dataset 3). Because of its potency, we selected the second-generation inhibitor Quisinostat as the primary compound for follow up experiments. The effect of Quisinostat on H1.0 levels was not restricted to breast cancer cells and treatment induced strong upregulation of H1.0 in a panel of 17 cell lines from 10 cancer types (Fig. 1d). Thus, Quisinostat widely restores high levels of H1.0 in cancer cells.

The HDACi identified in the screen are all broad-spectrum inhibitors that target multiple enzymes across various HDAC classes^20^. To identify specific enzymes that may be responsible for the effect on H1.0, we treated HCC1569 cells with HDACi specific for distinct HDAC classes (Entinostat: class I; PCI-34051: HDAC8; TMP195: class IIa; Tubastatin A: class IIb; Supplementary Dataset 3) and measured H1.0 levels, monitoring the acetylation of the core histone H3 and alpha-tubulin to confirm the compounds’ specificity. Tubastatin A and Entinostat, but not PCI-34051 and TMP195, induced a significant increase in H1.0 levels, although to a much lower extent compared with the effect observed upon Quisinostat treatment (Supplementary Fig. 2a–c). Only compounds increasing H3 acetylation affected H1.0 levels (Supplementary Fig. 2a, b). Knockdown of individual HDACs confirmed these results showing that downregulation of several class I and class IIb HDACs increased H1.0 levels (Supplementary Fig. 2a). We conclude that multiple HDACs regulate H1.0 expression and that their simultaneous inhibition is required to restore high H1.0 levels. Since H1.0 upregulation upon HDACi treatment correlated with H3 hyperacetylation, we compared the kinetics of Quisinostat-induced changes on H1.0 and acetylated core histones. H1.0 levels were elevated 2 h after Quisinostat addition and further increased by 24 h, with kinetics similar to those observed for global and residue-specific levels of acetylated core histones (Fig. 1e and Supplementary Fig. 2d). Thus, H1.0 begins to be re-expressed early upon Quisinostat treatment, following core histone hyperacetylation.

To characterize how HDACi treatment leads to an increase in H1.0 levels, we first examined whether H1.0 is acetylated. Western blot analysis of acid-extracted histones using an anti-acetyl-lysine antibody did not detect any acetylation in H1.0, neither in untreated or Quisinostat-treated cells, whereas acetylated core histones showed the expected increase upon treatment (Supplementary Fig. 2e). Mass spectrometry analysis confirmed these results, detecting numerous acetylated peptides from core histones but none from H1.0 in two distinct cell lines (Supplementary Fig. 2f). Thus, changes in protein acetylation are unlikely to underpin the increase in H1.0 levels induced by HDACi treatment. We then assessed whether H1.0 upregulation is due to transcriptional changes. Quantification of *H1F0* mRNA levels by qRT-PCR upon Quisinostat treatment revealed a progressive upregulation over 24 h, which mirrored the changes detected at the protein level (Fig. 1f). mRNA upregulation correlated with an increase in activating histone marks (H3K27ac and H3K9ac) at the *H1F0* promoter, suggesting that changes in core histone acetylation induced by Quisinostat promote transcription of the *H1F0* gene (Fig. 1g).

### Quisinostat inhibits cancer cell self-renewal in many cancers

**Fig. 2:**
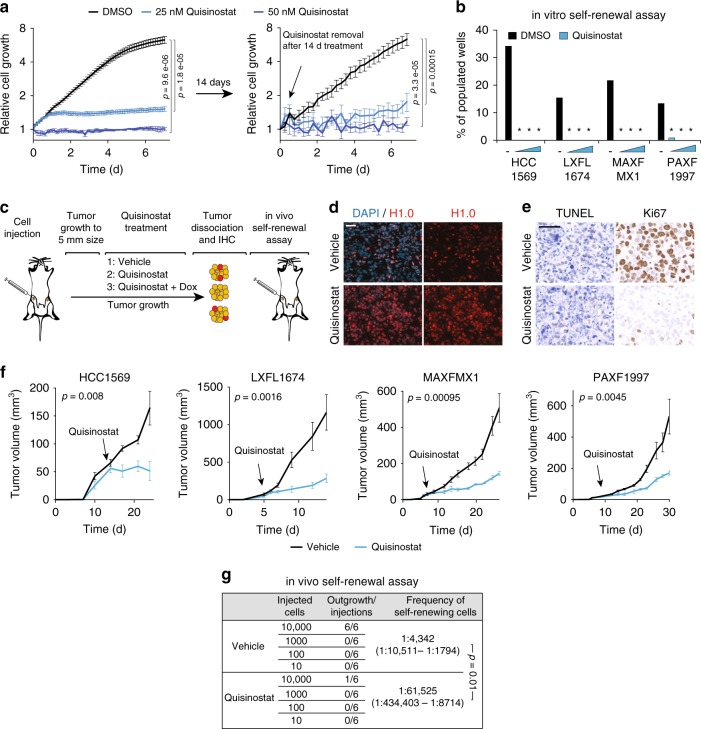
Quisinostat inhibits cancer cell self-renewal and drives differentiation. **a** IncuCyte proliferation assay on HCC1569 cells treated with Quisinostat for 7 days (left), or grown in the absence of the drug after a 14 d treatment. Values represent mean ± s.e.m. from four (left) or six (right) biological replicates. *p*-value: one-tailed Student’s *t*-test calculated at the last time point. TDF cells showed similar kinetics. **b** Clonogenic assays measuring self-renewal ability of the indicated cells. The percentage of wells (*n* = 96) populated by colonies is indicated. Cells were plated at limiting dilutions and treated with DMSO (black dash), or Quisinostat (12.5, 25, 50 nM, blue triangle). *p-value < 0.001 comparing respective DMSO- and Quisinostat-treated cells (two-way contingency table analysis and two-tailed Fisher’s exact test). Exact *p*-values in Source Data file. **c** Experimental design to assess the in vivo effect of Quisinostat. 1, 2 and 3 indicate different mice groups treated with the indicated substances. Group 3 relates to Fig. 4 where doxycycline (Dox) was used to induce H1.0 knockdown. IHC: immunohistochemistry. Red, yellow: self-renewing and non-self-renewing tumor cells, respectively. **d** Immunofluorescence microscopy of HCC1569-induced tumors. See also LXFL1674 in Supplementary Fig. 3g. Similar results were obtained in three tumors and with other PDXs. Scale bar: 50 µm. **e** Immunodetection of proliferating (Ki67^+^) and apoptotic (TUNEL^+^) cells by immunohistochemistry in treated tumors. Similar results were obtained in three tumors. Scale bar: 50 µm. **f** Tumor maintenance assay. Growth kinetics of HCC1569-induced tumors and the indicated PDXs treated with Quisinostat or vehicle. Arrow: start of Quisinostat treatment. Values are mean ± s.e.m. from four (HCC1569) or six tumors (PDX). *p*-value: one-tailed Student’s *t*-test calculated at the last time point. Three significant outlier tumors were excluded from the analysis (see source data). **g** Limiting-dilution transplantation assay into NSG mice using the indicated number of cells from Quisinostat- and vehicle-treated HCC1569-induced tumors. Despite the large confidence interval of the frequency estimate, due to the appearance of tumors at only one cell dose, differences are significant (*p* = 0.01 *χ*^2^ test). Data for all graph are in Source Data file.

We have previously shown that spontaneous, heterogeneous re-expression of H1.0 within tumors inhibits cancer cell self-renewal and creates functionally distinct subsets of cells: cells that stably repress H1.0 preserve self-renewal ability, whereas cells that reverse H1.0 silencing during tumor growth lose long-term proliferative capacity^16^. Furthermore, expression of exogenous H1.0 via genetic means inhibits cancer cell self-renewal and tumor maintenance^16^. As HDACi treatment induces strong upregulation of H1.0, we examined whether HDACi-treated cells showed impaired proliferative potential, using a variety of in vitro and in vivo assays. In agreement with previous reports, both HCC1569 and TDF cells were highly sensitive to both Quisinostat and Abexinostat in proliferation assays (Fig. 2a and Supplementary Fig. 3a). Although high compound doses (1 µM or higher) showed cytotoxicity, treatment with lower doses of compounds (25–50 nM for Quisinostat, 250–500 nM for Abexistonast) blocked cell proliferation without inducing substantial cell death (Fig. 2a and Supplementary Fig. 3a, b). Prolonged treatment for 14 days induced stable cytostasis even after drug removal, suggesting that cells had stably exited the cycle, consistent with a differentiation process (Fig. 2a). Analysis of surface markers further indicated that Quisinostat-treated HCC1569 cells were not just arrested, but had undergone a phenotypic transition, as CD44^+^CD24^−^ cells, a subpopulation shown to contain self-renewing tumor-propagating cells^26^, disappeared upon treatment (Supplementary Fig. 3c, d). In line with the observed phenotypic changes, Quisinostat-treated HCC1569 cells exhibited strongly impaired self-renewal ability in clonogenic assays (Fig. 2b), being unable to form mammospheres even at nanomolar concentration of the compound when seeded at limiting dilutions (Methods). These results were confirmed using patient-derived xenografts (PDXs) from multiple cancer types. Cells from breast (MAXFMX1), lung (LXFL1674) and pancreas (PAXF1997) cancer patients upregulated H1.0 upon Quisinostat treatment (Supplementary Fig. 3e) and displayed strongly inhibited self-renewal ability, independently of the basal frequency of clonogenic cells in the population (Fig. 2b and Supplementary Fig. 3f). Thus, self-renewing cells from various cancer types are sensitive to Quisinostat treatment.

In vivo assays corroborated and strengthened these findings (Fig. 2c–g). Quisinostat treatment of established tumors induced by orthotopic injection of HCC1569 cells into NOD.Cg-Prkdc^scid^Il2rg^tm^1Wjl/SzJ (NSG) mice restored homogeneously high H1.0 levels and effectively halted tumor growth (Fig. 2c–f). We also observed strong inhibition of tumor maintenance in the three PDX models of lung, pancreas and breast cancer (Fig. 2f). Treated tumors showed reduced fractions of proliferating Ki67^+^ cells and no increase in apoptotic TUNEL^+^ cells, confirming that Quisinostat treatment induced cytostasis, not cell death, in vivo (Fig. 2e). We then examined whether the anti-tumor effect of Quisinostat affected self-renewing tumor cells. In agreement with our previous observations, although H1.0 was overall downregulated within tumors, self-renewing cells isolated from PDXs as spheroids expressed particularly low levels of *H1F0*, confirming an inverse relationship between cancer cell self-renewal and H1.0 levels (Supplementary Fig. 3g). Of note, no self-renewing cells could be isolated from HDACi-treated PDXs, which expressed homogeneously high H1.0 levels (Supplementary Fig. 3g). To directly assess the effect of Quisinostat on cancer cell self-renewal in vivo, we performed limiting-dilution transplantation assays for secondary tumor formation (Fig. 2c). Quisinostat-treated tumors contained ~14-fold less tumor-propagating cells compared with vehicle-treated tumors, indicating strong inhibition of cancer cell self-renewal (Fig. 2g). We conclude that Quisinostat treatment restores homogeneous high levels of H1.0 within tumors and inhibits tumor maintenance by impairing both the short- and the long-term proliferative potential of cancer cells.

**Fig. 3:**
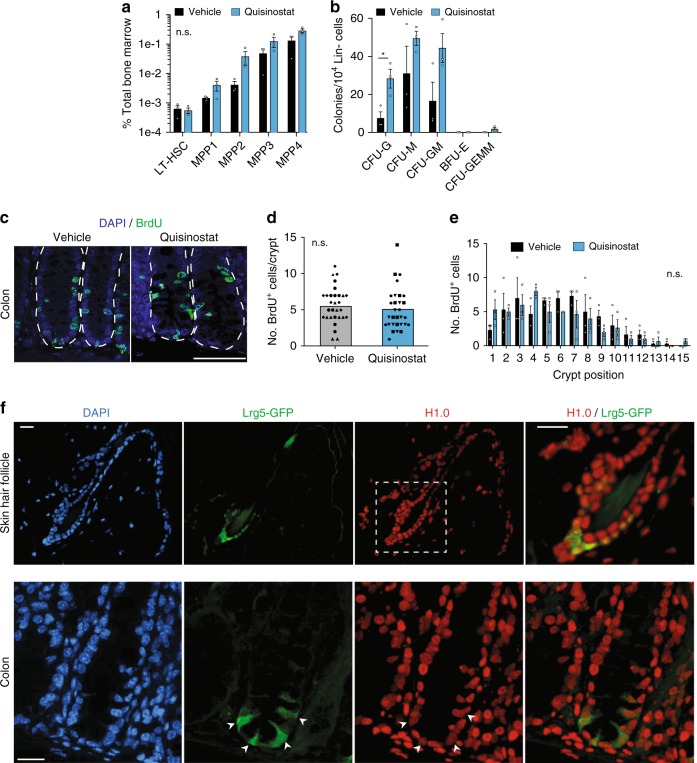
Quisinostat treatment does not impair normal tissue stem cell function. **a**, **b** Relative abundance of long-term hematopoietic stem cells (LT-HSCs) and multipotent progenitors 1–4 (MPPs 1–4) in the bone marrow of vehicle- or Quisinostat-treated mice as assessed by flow cytometry (**a**) or colony assay (**b**). Colony-forming units (CFU) per 10,000 lineage negative (Lin−) cells extracted from femurs of vehicle- or Quisinostat-treated mice and plated in methylcellulose for 7 days (**b**). CFU-G (granulocyte), CFU-M (macrophages), CFU-GM (granulocyte/macrophages), BFU-E (Burst-Forming Unit-Erythroid), CFU-GEMM (Granulocyte/Erythrocyte/Macrophage/Megakaryocyte). Values are mean ± s.e.m. from three mice per treatment condition. The differences between treated and untreated cells for each cell type in both panels is not significant (n.s. *p* > 0.05) except for CFU-G, where *p = 0.02, (two-tailed Student’s *t*-test). **c** Immunodetection of dividing cells (BrdU^+^, green) in the colon crypts in Quisinostat- or vehicle-treated mice. Cell nuclei are marked by DAPI (blue). Dotted lines indicate crypts. Scale bars: 100 μm. **d** Quantification BrdU^+^ cells per colon crypt in Quisinostat- or vehicle-treated mice. Each point represents an individual crypt from three mice, ten crypts analyzed per mouse, with different symbols indicating crypts from different mice. Bars indicate the median value. n.s.: non-significant (*p* = 0.4199, unpaired two-tailed Students’ *t*-test). **e** Distribution of BrdU-positive cells across crypt positions in Quisinostat- or Vehicle-treated mice. Position 1 represents the first cell in crypt away from the midline. Values represent mean ± s.e.m. from three mice, with ten crypts quantified for each mouse. n.s.: non-significant (*p* = 0.5346, two-way ANOVA). **f** Immunodetection of H1.0 in colon and hair follicle stem cells marked by Lrg5. Tissues are from transgenic mice expressing GFP under the control of the endogenous Lrg5 promoter^28^. Scale bars: 20 μm. For the skin hair follicle, the area in the white square is shown at a higher magnification as merge. For the colon, the white arrows indicate Lrg5^+^ cells. Similar patterns were observed in three sections. Data for all graphs is in Source Data file.

In line with the good safety profile of Quisinostat in patients^18,19^, mice treated with the drug did not suffer evident side effects or weight loss (Supplementary Fig. 4a). In agreement, treatment did not impair normal tissue stem cell function, as revealed by analysis of various tissues characterized by high cellular turnover (Fig. 3a–e and Supplementary Fig. 4b). We found similar fractions of hematopoietic stem and progenitor cells (see Methods for specific markers) in the bone marrow in vehicle- and Quisinostat-treated mice, and their colony-forming ability was either unaffected or in fact enhanced by the drug (Fig. 3a, b). Furthermore, skin hair follicles of all Quisinostat-treated mice showed efficient BrdU incorporation, indicating normal cell proliferation within the bulge compartment, where epidermal stem cells reside (Supplementary Fig. 4b). Similarly, colon crypts displayed very similar patterns of BrdU^+^ cells in control and Quisinostat-treated mice, with a gradient of labeled cells from the stem and progenitor cell zone at the base to the differentiated cell region up the crypts, indicating normal tissue maintenance (Fig. 3c–e). Of note, expression of H1.0 was readily detected in cells marked by the stem cell marker Lrg5^+^^27,28^, both in hair follicles and in colon crypts, indicating that H1.0 expression is not detrimental for normal self-renewing cells (Fig. 3f). Thus, Quisinostat specifically impairs cancer cell self-renewal without affecting normal stem cell function.

### Quisinostat acts primarily through H1.0

**Fig. 4:**
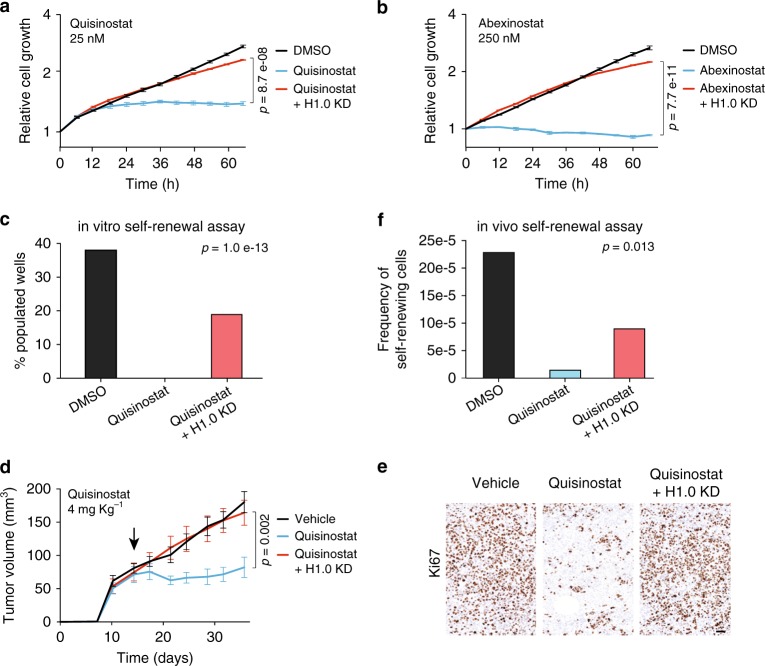
Quisinostat inhibits cancer cell self-renewal by restoring high H1.0 levels. **a**, **b** IncuCyte proliferation assay on HCC1569 cells treated with the indicated HDACi. HCC1569 cells used in these experiments contain an inducible H1.0-targeting shRNA (shH1.0_1), which is either not expressed (Quisinostat/Abexinostat) or expressed to prevent H.10 upregulation (Quisinostat/Abexinostat + H1.0 KD). See also Supplementary Fig. 5e, h (independent H1.0-targeting shRNA and a non-targeting shRNA). Values represent mean ± s.e.m. from four biological replicates. Similar results were obtained in three independent experiments. *p*-value: one-tailed Student’s *t*-test calculated at the last time point. **c** Clonogenic assays measuring self-renewal ability of HCC1569 cells. The percentage of wells (*n* = 96) populated by colonies is shown. Cells contain an inducible H1.0-targeting shRNA (shH1.0_1) which is not expressed (Quisinostat) or expressed to prevent H1.0 upregulation (Quisinostat + H1.0 KD). Cells were treated with 12.5 nM Quisinostat with (H1.0 KD) or without 0.5 µg ml^−^^1^ Doxycycline. With DMSO-treated H1.0 KD cells, the percentage of populated wells was 39.6%. The significance of the overall difference among the conditions is indicated (three-way contingency table analysis and two-tailed Fisher’s exact test). **d** Tumor maintenance assay. Growth kinetics of HCC1569-induced tumors treated with 4 mg kg^−1^ Quisinostat or vehicle. Injected cells contain an inducible H1.0-targeting shRNA (shH1.0_1) which is either not expressed (Quisinostat) or expressed to prevent H1.0 upregulation (Quisinostat + H1.0 KD). Values represent mean ± s.e.m. from eight tumors. *p*-value: one-tailed Student’s *t*-test calculated at the last time point. Arrow: start of treatment. Similar results were obtained in an independent experiment. See also Supplementary Fig. 5k. **e** Immunodetection of proliferating (Ki67^+^) cells by immunohistochemistry in one of three tumors per condition. Scale bar: 50 µm. **f** Limiting-dilution transplantation assay into NSG mice using cells from HCC1569-induced tumors treated with the indicated substances. The significance of the overall difference among the conditions is indicated (*χ*^2^ test). *n* = 6 tumors. Outgrowths/injections for H1.0 KD: 4/6, 0/6, 0/6 and 0/6 for 10,000, 1000, 100, 10 injected cells, respectively. For the other conditions, see Fig. 2g. Data for all graphs in Source Data file.

HDACs are abundant epigenetic modifiers that regulate a variety of proteins and histone modifications present throughout the genome. Thus, inhibition of their activity is likely to affect several cellular factors and have pleiotropic effects. To quantify the contribution of H1.0 to mediating the anti-tumor effect of HDACi, we introduced a dox-inducible H1.0-targeting shRNA (shH1.0_1) in HCC1569 and TDF cells to prevent H1.0 re-expression upon treatment (Supplementary Fig. 5a). Induction of shH1.0 efficiently counteracted the effect of multiple HDACi, maintaining H1.0 levels similar to those measured in control cells treated with DMSO (Supplementary Fig. 5a, b). H1.0 knockdown did not induce compensatory changes in other histone H1 variants and did not substantially alter the acetylation status of core histones upon HDACi treatment (Supplementary Fig. 5c, d). We then examined the effect of Quisinostat or Abexinostat on the proliferative potential of cells unable to re-express H1.0, using both HCC1569 and TDF cells. For this analysis, the lowest dose found to halt cell proliferation and self-renewal was used for each compound. In all cases, H1.0 knockdown, but not induction of a non-targeting shRNA, effectively rescued the proliferation of HDACi-treated cells, leading to growth kinetics very similar to those of control cells (Fig. 4a, b and Supplementary Fig. 5e–g). Cells expressing a distinct H1.0-targeting shRNA (shH1.0_2), or cells in which *H1F0* had been knocked-out by CRISPR-mediated gene editing, showed a similarly efficient rescue of proliferation (Supplementary Fig. 5a, h–j). H1.0 knockdown also counteracted the effect of Quisinostat in clonogenic assays, indicating that H1.0 re-expression is primarily responsible for the observed self-renewal inhibition (Fig. 4c). More importantly, the anti-tumor effect of Quisinostat in vivo was entirely abrogated when H1.0 re-expression was prevented by induction of shH1.0_1, and growth rescue was observed in three independent experiments using different drug dosages (Fig. 4d and Supplementary Fig. 5k). Quisinostat-treated, H1.0-knocked-down tumors contained fractions of Ki67^+^ cells comparable to vehicle-treated tumors and limiting-dilution transplantation assays showed a rescue of cancer cell self-renewal in vivo (Fig. 4e, f). Taken together, these results demonstrate that Quisinostat impairs cancer cell self-renewal and tumor maintenance primarily by restoring high levels of H1.0.

### Self-renewal-associated oncogenic pathways regulated by H1.0

**Fig. 5:**
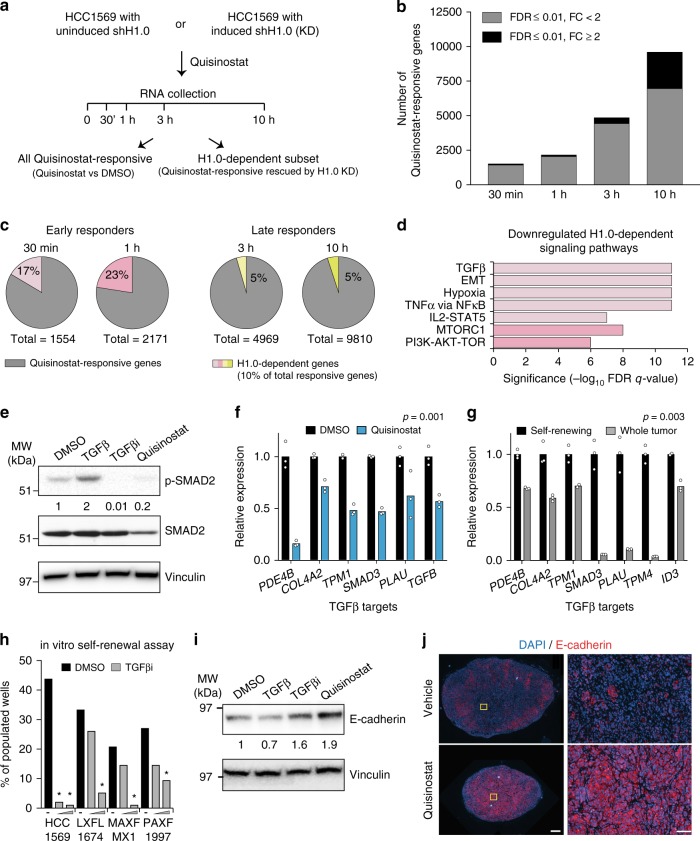
A minority of Quisinostat-responsive genes are responsible for inhibiting cancer cell self-renewal. **a** Experimental design of the gene expression analysis. **b**, **c** Quantification of Quisinostat-responsive genes (**b**) and the subset of H1.0-dependent genes (**c**) at the indicated time points. FDR: false discovery rate. FC: fold-change. **d** Signaling pathways downregulated by Quisinostat treatment and rescued by H1.0 knockdown, as assessed by GSEA analysis. Gene signatures downregulated at 30 min (light pink) and at 1 h (dark pink) are shown. See also Supplementary Fig. 6c. **e** Quantification of total and phosphorylated SMAD2 (p-SMAD2) in HCC1569 cells by western blot analysis. Vinculin: loading control. Treatments with recombinant TGFβ (TGFβ) or the TGFβ inhibitor SB431542 (TGFβi) were performed as controls. Similar results were obtained in an independent experiment. **f**, **g** qRT-PCR analysis of the indicated TGFβ target genes^53^ in Quisinostat-treated HCC1569 cells (**f**), or in self-renewing cells isolated as spheroids from LXFL1674 PDX and the corresponding whole tumor (**g**). Values are mean from three technical replicates. The statistical significance of the overall difference, indicative of differential pathway activity, is indicated (paired two-tailed Student’s *t*-test). **h** Clonogenic assays measuring self-renewal ability of the indicated cells. The percentage of wells (*n* = 96) populated by colonies is indicated. Black dash: DMSO; gray triangle: TGFβi. *p < 0.05 (two-way contingency table analysis and two-tailed Fisher’s exact test). Exact *p*-values in Source Data file. **i** Quantification of E-cadherin in HCC1569 cells by western blot analysis. Vinculin: loading control. Treatments with recombinant TGFβ (TGFβ) or the TGFβ inhibitor SB431542 (TGFβi) were performed as controls. Note the stronger effect of Quisinostat compared to TGFβi suggesting that additional signaling pathways promoting EMT are affected by Quisinostat. Similar results were obtained in an independent experiment. **j** Immunofluorescence microscopy of HCC1569-induced tumors treated with Quisinostat and control tumors (vehicle) detecting E-cadherin (red). Panels on the right are enlargements of the area marked in yellow on the left. Similar results were obtained in three tumors per condition and in the three PDXs. Scale bar: 500 µm left panels, 100 µm right panels. Data for all graphs are in Source Data file.

H1.0-knocked-down cells, which are insensitive to HDACi treatment, offer an opportunity to uncouple transcriptional programs responsible for inhibition of cancer cell self-renewal from other HDACi-induced gene expression changes. To do so, we performed RNA-seq analysis of HCC1569 cells at various time points after Quisinostat treatment, allowing or preventing H1.0 re-expression (Fig. 5a, Supplementary Fig. 6a, d). At each time point, we searched for genes affected by Quisinostat (FDR ≤ 0.01) relatively to the DMSO-treated control cells, and rescued in their expression by H1.0 knockdown (FDR ≤ 0.01). Quisinostat treatment induced detectable changes in gene expression by 30 min and a progressive increase in the number of differentially expressed genes (DEGs) over time, reaching ~10,000 by 10 h (Fig. 5b and Supplementary Fig. 6b). Most changes were moderate in magnitude (fold-change <2), indicating that Quisinostat treatment induces subtle but widespread transcriptional changes across the genome (Fig. 5b). Only 10% of all Quisinostat-responsive genes were rescued by H1.0 knockdown, indicating a role for these genes in self-renewal inhibition (Fig. 5c). Most H1.0-dependent genes were observed among early responders (30 min and 1 h), suggesting that the late transcriptional response is largely unrelated to the effect on cell self-renewal (Fig. 5c). Gene set enrichment analysis (GSEA)^29^ of H1.0-dependent genes revealed significant downregulation of several gene signatures starting at 30 min (FDR ≤ 10^−6^), including TGFβ signaling, NFκB activation, STAT signaling and epithelial-to-mesenchymal transition (EMT), followed by downregulation of MYC targets and mTOR signaling at 1 h (Fig. 5d and Supplementary Fig. 6c). Of note, many of the identified pathways have been implicated in the maintenance of cancer cell self-renewal^30–33^. Later time points showed downregulation of E2F targets and other cell cycle-related genes, in line with the observed decrease in cell proliferation (Supplementary Fig. 6c). H1.0-dependent late responders included *CDKN1A*/p21, an established HDACi-induced gene (Supplementary Fig. 6d). Thus, multiple oncogenic pathways that sustain cancer cell self-renewal are regulated by H1.0 and are simultaneously inactivated upon Quisinostat treatment.

To confirm the GSEA results, we focused on the TGFβ pathway and the EMT process, because of their established link^34^. Quisinostat treatment reduced the levels of phosphorylated SMAD2, a major transducer of TGFβ signaling, with comparable efficiency as an inhibitor blocking TGFβ type I receptors (TGFβi, SB431542) (Fig. 5e). Lower levels of phosphorylated SMAD2 in Quisinostat-treated cells were due to a decrease in total SMAD2 levels, indicating that Quisinostat inhibits the TGFβ pathway partly by decreasing the total amount of this key signal transducer (Fig. 5e). Confirming pathway inactivation, several TGFβ target genes were downregulated in multiple models upon treatment (Fig. 5f and Supplementary Fig. 6e). In line with a role for TGFβ signaling in sustaining cancer cell self-renewal, self-renewing cells isolated from PDXs displayed higher expression of TGFβ targets compared to the tumor bulk, indicative of pathway activation, and showed reduced clonogenic ability upon signaling inhibition by TGFβi (Fig. 5g, h). Furthermore, Quisinostat treatment increased the levels of E-cadherin, both in vitro and in vivo, indicating a reversion to a more epithelial phenotype (Fig. 5i, j and Supplementary Fig. 6f). Notably, numerous H1.0-regulated oncogenic pathways identified by GSEA favor the EMT process^34,35^, suggesting that their simultaneous inactivation by Quisinostat converges into promoting a more differentiated, epithelial phenotype. The mechanistic basis of how H1.0 represses self-renewal-sustaining genes by stabilizing nucleosome-DNA interactions at their promoters has previously been uncovered^16^.

### The Quisinostat–H1.0 axis inhibits disease relapse

**Fig. 6:**
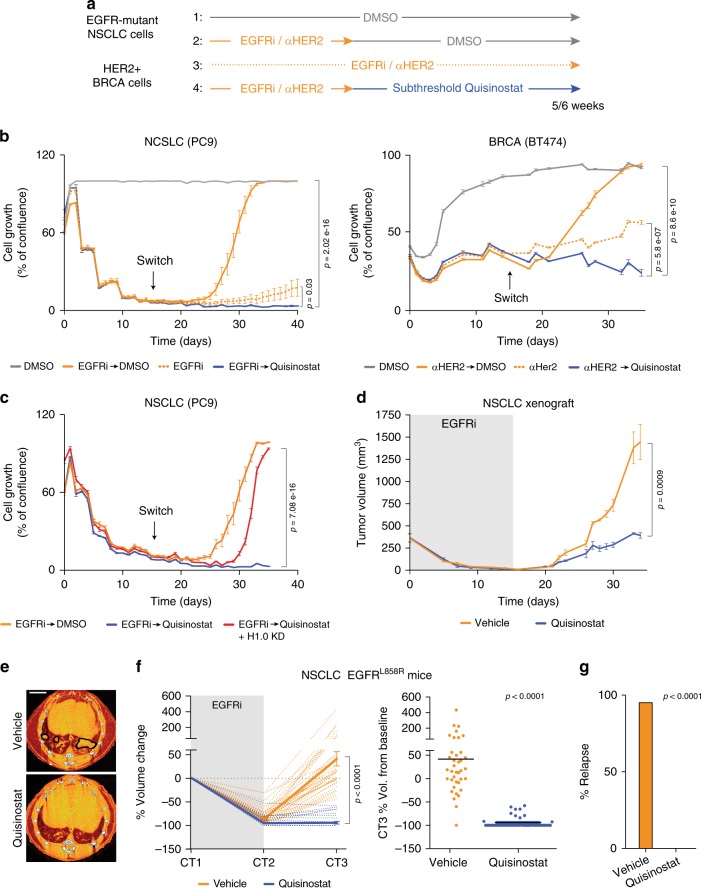
Quisinostat treatment inhibits expansion of cells surviving targeted cytotoxic agents. **a** Experimental design to assess the effect of Quisinostat on cells surviving EGFRi and anti-HER2 antibody (αHER), showing conditions tested in **b**. Subthreshold Quisinostat dose: 10 nM after 14 days of targeted therapy (switch). **b** IncuCyte proliferation assay on EGFR-mutant non-small lung cancer (NSCLC) PC9 cells and breast invasive carcinoma (BRCA) BT474 cells. The line steps correspond to media change time points. Values represent mean ± s.e.m. from five biological replicates. Similar results were obtained in multiple experiments. *p*-value calculated at the last time point (one-tailed Student’s *t*-test). **c** IncuCyte proliferation assay on PC9 cells containing an inducible H1.0-targeting shRNA (shH1.0_1) which is either not expressed (Quisinostat) or expressed to prevent H1.0 upregulation (Quisinostat + H1.0 KD). Values represent mean ± s.e.m. from five biological replicates. *p*-value calculated at the last time point (one-tailed Student’s *t*-test). **d** Growth kinetics of tumors induced by injection of EGFR-mutant NSCLC H1975 cells. Sequential treatment with osimertinib (gray area) and Quisinostat or vehicle. Values represent mean ± s.e.m. from four tumors. *p*-value calculated at the last time point (one-tailed Student’s *t*-test). Similar results were obtained in two independent experiments. **e** Representative CT scans (CT3) of EGFR^L858L^ mice treated with vehicle or Quisinostat after EGFRi treatment (Erlotinib) and quantified in **f**. Black lines: detected tumors. Orange areas: bronchi and blood vessels. Scale bar: 4 mm. **f** Response to sequential EGFRi-Quisinostat therapy in EGFR^L858L^ mice, expressed as percentage of tumor volume change relative to the start of treatment (left) and at endpoint (right). Sequential treatment with EGFRi (gray area) and Quisinostat or vehicle. CT scans performed at 1 month intervals. Dotted lines: individual tumors; solid lines: average values ±s.e.m. (left). Each dot is an individual tumor; black line: mean value (right). *n* = 38 for vehicle, 41 for Quisinostat. *p*-value: two-tailed Mann–Whitney test. Vol: Volume. **g** Percentage of relapsed tumors at endpoint. *n* = 31 for vehicle and 21 for Quisinostat. Only tumors undetectable at CT2 are scored. *p*-value: two-way contingency table analysis and two-tailed Fisher’s exact test. Data for all graphs are in Source Data file.

In addition to being critical for tumor maintenance in steady-state conditions, cancer cell self-renewal is also responsible for disease relapse when cells survive drug treatment: if surviving cells do not have self-renewal capacity, they cannot reconstitute the tumor. The ability to regenerate the tumor mass is distinct from the ability to survive treatment and, independently of the resistance mechanism, it is required for disease recurrence. We therefore asked whether inhibition of self-renewal by the Quisinostat–H1.0 axis could inhibit disease relapse upon failure of cytotoxic targeted therapy. To do so, we selected, among cell lines found to re-express H1.0 upon Quisinostat treatment (Fig. 1d), lines from different cancer types that are known to develop acquired resistance to targeted agents: non-small lung cancer (NSCLC) cells that model resistance to EGFR tyrosine kinase inhibitors (EGFRi) in EGFR-mutated patients (PC9, HCC827 and H1975, cell line 13, 14, 15 in Fig. 1d) and breast cancer cells that develop resistance to the anti-HER2 antibody Trastuzumab (BT474, cell line 16 in Fig. 1d). Despite the excellent response typically observed in patients, targeted therapy is not curative and patients almost invariably relapse^36,37^. In agreement with previous reports that used different compounds^38^, we observed that both breast cancer and lung cancer cell lines contained cells surviving the targeted agents (Supplementary Fig. 7a and Fig. 6a, b). These cells remained in a quiescent state over the course of treatment, but upon drug removal, they resumed proliferation and reconstituted an expanding cell population, mimicking the behavior observed in patients (Fig. 6a, b and Supplementary Fig. 7a, b). To investigate a possible synergy between the Quisinostat–H1.0 axis and the targeted agents, after 2 weeks of treatment with EGFRi/anti-HER2 antibody, we removed the drugs and treated surviving cells with Quisinostat for an additional 2–3 weeks (Fig. 6a–c and Supplementary Fig. 7a, b). Quisinostat treatment prevented reconstitution of an expanding cell population and, importantly, was more effective than continuous treatment with EGFRi/anti-HER2 antibody (Fig. 6a, b). Moreover, cells surviving EGFRi/anti-HER2 antibody failed to re-enter the cell cycle at subthreshold Quisinostat concentrations that did not affect cycling cells untreated with the targeted agents (Supplementary Fig. 7b). This observation suggests preferential sensitivity of surviving cells to the inhibiting effect of Quisinostat, possibly favored by their transient quiescent state. Although the Quisinostat effect was initially cytostatic, a gradual reduction in the number of surviving cells was observed over 3 to 4 weeks, suggesting that differentiated cells progressively died (Fig. 6b, c and Supplementary Fig. 7b). The response to HDACi was abrogated in cells which could not restore high H1.0 levels due to the expression of shH1.0_1, indicating that H1.0 is primarily responsible for the observed effect (Fig. 6c). Although all tested HDACi showed activity in the sequential treatment setting, only Quisinostat elicited a durable growth inhibition (Fig. 6b and Supplementary Fig. 7b), while Vorinostat and Abexinostat only showed a transient effect despite the higher dose used (Supplementary Fig. 7c). Thus, Quisinostat is not only more potent but also more effective than the first-generation HDACi commonly used in patients.

To extend the analysis in vivo, we focused on EGFRi-resistant NSCL cancer, as relevant mouse models are available and have been characterized. We initially assessed the efficacy of the sequential drug treatment in a xenograft model and observed a strong inhibition of tumor relapse in Quisinostat-treated mice (*p* < 0.001) (Fig. 6d). We then employed an autochthonous mouse model that develops spontaneous lung tumors upon activation of an EGFR^L858R^ allele (EGFR^L858R^ mice)^39^ (Fig. 6e–g). Treatment of established tumors with EGFRi led to efficient tumor regression, with numerous nodules becoming undetectable after one month of treatment, as assessed by micro computerized tomography (μCT) (Fig. 6f and Supplementary Fig. 7d). However, upon interruption of EGFRi administration, 95% of seemingly eliminated tumors reappeared and within a month reached sizes comparable to that measured at the start of treatment (Fig. 6e–g and Supplementary Fig. 7d). In contrast, all regressed tumors undergoing sequential treatment with Quisinostat remained undetectable, and those that showed incomplete response to EGFRi grew significantly less than control tumors (Fig. 6e–g and Supplementary Fig. 7d). Similar results were obtained with EGFR^L858R^ mice lacking p53 (Supplementary Fig. 7e). Thus, sequential administration of a targeted agent that kills most bulk tumor cells and Quisinostat that prevents expansion of surviving cells induces a durable response in mice.

## Discussion

Our findings identify a well-tolerated compound^18,19^ as an effective inhibitor of cancer cell self-renewal, which halts tumor maintenance and inhibits expansion of cells surviving targeted cytotoxic agents. Despite the availability of small molecules targeting uncontrolled self-renewal through interference with signaling pathways such as Wnt, Notch and Hhg, the utility of these compounds has been limited by their non-specific effect and general toxicity^14^. As normal stem cells heavily rely on the same pathways, finding a therapeutic window has been difficult. In contrast, the established safety of HDACi suggests specific targeting of cancerous self-renewing cells. Indeed, we show that Quisinostat does not impair normal stem cell function, similarly to what previously reported for other HDACi^40–42^. This observation, together with the finding that H1.0 is highly expressed in tissue stem cells, indicates that distinct epigenetic mechanisms control self-renewal of normal and malignant cells, making Quisinostat an attractive means to disable the cells that fuel the long-term cancer growth and drive disease relapse. The specific effect of HDACi on malignant self-renewing cells is likely due to the fact that cancer cells have a severely altered epigenome and respond differently to epigenetic challenges compared with normal cells^43,44^.

Our results suggest that clinical efficacy of Quisinostat, and of HDACi in general, should not be measured by assessing tumor regression, since the drugs are not cytotoxic in vivo but rather deprive cells of long-term proliferative capacity. Such a mechanism of action requires time to show its effect and only affects a subset of cancer cells—those that self-renew, which typically represent a minority of the tumor cell population. Thus, parameters such as long-lasting disease stabilization or progression free-survival are better indicators of their potential effect. Notably, this is the effect commonly observed in patients^18,45^. Importantly, our data show that the second-generation inhibitor Quisinostat, characterized by increased potency and bioavailability^17^, elicits stronger and more durable effects than Vorinostat, the most commonly used HDACi in patients, suggesting that the clinical efficacy of HDACi may have been considerably underestimated.

While efficient inhibition of tumor maintenance could be, in principle, curative in the long-term, since cancer cells spontaneously dying would not be replenished in the absence of self-renewing cells, cytostatic therapy is unlikely to be fully effective on its own. However, combining cytotoxic treatments that kill most tumor cells and cytostatic agents that prevent expansion of surviving cells may be an effective strategy to achieve a durable response in patients. Indeed, we show that administration of Quisinostat after targeted therapy blocks expansion of surviving cells in two distinct models of acquired resistance in vitro, and strongly inhibits lung cancer relapse in EGFR^L858R^ mice. In agreement with the response observed in patients, virtually all EGFR^L858R^-driven tumors that initially responded to EGFRi contained resistant cells that eventually led to therapy failure. However, these cells were unable to reconstitute the tumors when mice were treated with Quisinostat. This finding represents a proof of principle that combining complementary approaches that target bulk tumor cells and self-renewing surviving cells can be highly effective. Importantly, such an approach does not require any knowledge of the mechanism of resistance, which is typically highly diverse across patients^46^, since it acts on the second step of disease relapse: the reconstitution of the cancerous mass. Thus, while combination therapies that seek to exploit specific vulnerabilities of resistant cells are only suitable for patients showing a particular resistance mechanism, the potential benefit of Quisinostat is independent of how resistance emerges.

Identification of H1.0 as a major mediator of the cytostatic effect of Quisinostat suggests broad utility of this compound in cancer, since H1.0 is commonly downregulated and is an independent predictor of patient outcome in numerous cancer types^16^. Moreover, while cancer drivers and the associated resistance to targeted therapy are often cancer type-specific, self-renewal of resistant cells is a universal mechanism underpinning disease relapse. Thus, targeting cancer cell self-renewal through the Quisinostat–H1.0 axis may offer a tumor-agnostic approach to safely interfere with cancer progression and recurrence.

## Methods

### Tissue culture and constructs

Cell lines used in the study and respective growth conditions are listed in Supplementary Table 1. Inducible cell lines were generated by introducing a lentiviral pTRIPZ construct expressing an H1.0-targeting shRNAs (shH1.0_1: V2THS_38052, shH1.0_2: V2THS_38055, Open Biosystems)^16^. Both shRNAs have been extensively characterized previously and shown to have specific effects on H1.0 levels, expression of self-renewal genes, tumor maintenance and cancer cell self-renewal^16^. A control pTRIPZ plasmid expressing the mir30 cassette, rtTA3 and TurboRFP but no targeting shRNA was used as a negative control (non-targeting sequence: 5′-ATCTCGCTTGGGCGAGAGTAAG-3′). Virus production was performed by transfecting 293-T cells with the pTRIPZ constructs, psPAX2 and pMD2.G plasmids (Addgene) using Fugene HD (Promega). Cells infected with targeting and non-targeting shRNA constructs were isolated by cell sorting based on TurboRFP expression after transient induction with 1 μg ml^−1^ Doxycycline (dox) for 16 h (TDF) or selected with 1 μg ml^−^^1^ puromycin for 7 days (HCC1569, PC9). Homozygous H1.0-KO TDF cells were generated by CRISPR-mediated gene editing using two sgRNAs flanking the *H1F0* CGI shore and have been described^16^. Two distinct clones containing a 184 bp deletion starting 176 nucleotides after the first coding ATG were used. All cancer cell lines have been sourced from the Crick Institute common repository, authenticated by STR profiling and tested for mycoplasma.

### High-throughput screening

High-throughput primary screening was performed in triplicates using HCC1569 and TDF cell lines and a small molecule collection assembled from a number of commercial sources (Sigma, Selleck, Enzo, Tocris, Calbiochem and Symansis), comprising a total of 4239 well-characterized drug-like compounds. Compounds were tested at a 10 µM in 384-well CELLSTAR® cell culture microplates (Greiner Bio-One International 781091) and quantitative immunofluorescence microscopy was performed to measure H1.0 levels. 800 cells were seeded per well in 40 µl of RPMI containing 10% FBS, using a Xrd-384 reagent dispenser (FuidX, also used for all subsequent liquid handling steps unless otherwise indicated). Cells were incubated at 37 °C and 5% CO_2_ overnight. 40 nl of 10 mM compound stocks in DMSO were transferred to the wells using an Echo® 550 (Labcyte). 40 nl of DMSO (0.1% final concentration) were added to specific wells on each plate as a negative control, using the peri-pump dispenser of a EL406 dispenser washer (BioTek). Multiple DMSO control wells were included on each plate. After compound addition, plates were spun for 1 min at 189×*g* and returned to the incubator at 37 °C and 5% CO_2_. After 24 h of treatment, cells were fixed by the addition of 40 µl of 7.4% formaldehyde in PBS to a final concentration of 3.7% and incubated at room temperature for 30 min. After fixation, cells were washed three times with PBS using ELx405 washers (BioTek, also used for all subsequent washing and aspiration steps). For staining, PBS was first aspirated and cells are permeabilized by adding 20 µl PBS + 3% BSA + 0.5% Triton X-100 for 20 min. Immunofluorescence staining was performed by first incubating cells for 1 h in blocking buffer (PBS + 3% BSA + 0.05% Triton X-100), followed by 1 h incubation with a primary rabbit monoclonal H1.0 antibody (1:5000 dilutions of a 2.4 mg ml^−^^1^ custom batch, ab134914), washed three times with PBS, and then a 1 h incubation with a Donkey Anti-Rabbit AlexaFluor® 647 (Invitrogen A31573) diluted 1:1000 in the presence of 1 μg ml^−^^1^ DAPI (Roche 10236276001) in blocking buffer. Cells were finally washed three times with PBS before automated image acquisition of six fields per well was performed using a ×10 objective on a Cellomics Array Scan VTI (ThermoFisher Scientific). Image analysis was performed using HCS Studio v6.6.1 (Thermo Scientific).

### Screening data analysis

H1.0 signal intensity (Mean_Circ_Average_Intensity) in compound-treated wells was compared with the intensity measured in control wells. Sample values were first expressed as a percentage of the average DMSO control on their respective plate. The median value of the replicates for each plate was then calculated. For the HCC1569 screen, an additional smoothing factor was applied to account for positional effects. The threshold for active compounds was set at 140 for HCC1569 cells and 120 for TDF (Supplementary Dataset 1, 2).

### Validation of primary hits

Compounds used to validate the primary hits were purchased at 10 mM concentration in DMSO from Insight Biotechnology: PCI-24781 (HY-10990-1ml), JNJ-26481585/Quisinostat (HY-15433-1ml), MK2206 (HY-10358-1ml), WZ4002 (HY-12026-1ml), Mitoxantrone (HY-13502A-1ml), Ceftiofur (HY-B0898-1ml), Ozagrel (HY-B0428A-1ml), Vorinostat (HY-10221-1ml), Inauhzin (HY-15869-1ml), Atazanavir (HY-17367A-1ml), GSK3787 (HY-15577-1ml), Enzastaurin (HY-10342-1ml), IPI-145 (HY-17044-1ml), Leucovorin (HY-13664-1ml), SGI-1027 (HY-13962-1ml), Idarubicin (HY-17381-1ml) or from Sigma: Trichostatin A, Ready Made Solution (5 mM, T1952-200UL), Daunorubicin (30450-5MG, diluted to 10 mM in DMSO). For validation experiments, 6 × 10^3^ (HCC1569, HCC1954) or 3.5 × 10^3^ (all other cell lines) cells were plated in a 96-well imaging microplate (Falcon, 353219) and incubate at 37 °C and 5% CO_2_ overnight. Cells were then treated with increasing concentrations (1 nM, 10 nM, 100 nM, 1 µM, 3 µM and 10 µM) of each compound for 24 h in quadruplicates and H1.0 expression levels assessed by quantitative immunofluorescence microscopy as described above. DMSO and TSA (100 nM and 1 µM)-treated cells were included in each plate as negative and positive controls, respectively. Similar experiments were performed with specific HDACi, used to identify HDACs responsible for the increase on H1.0: Tubastatin A (A8547-1ml), Entinostat (A8171-1ml), PCI-34051 (A4091-1ml), TMP195 (HY-18361-1ml), purchased from Generon at 10 mM concentration in DMSO. Stained plates were imaged and images quantified as described above. More than 1000 cells were quantified in each well.

### Patient-derived-xenografts (PDX)

Information about the LXFL1674, MAXFMX1 and PAXF1997 PDX models are listed in Supplementary Table 2. Models were obtained from the Charles Rivers tumor model compendium https://compendium.criver.com/compendium2/cancertype?species.name=Human.

Self-renewing cells were isolated by plating dissociated tumor cells (see in vivo self-renewal assay section for details about dissociation) on uncoated plates (sterilin petri dishes, Thermo scientific, 101R20) in RPMI medium, refreshing the medium every 3 days until spheroids formed.

### Protein immunodetection

Antibodies used for immunofluorescence staining of cultured cells were: anti-H1.0 (abcam custom batch, ab134914, 1:5000), mouse anti-H1.0 (clone 3H9^47^, 1:1000), rabbit monoclonal [EPR16606] anti-acetylated Histone H4 (acetyl K5 + K8 + K12 + K16) (abcam, ab177790, 1:5000), rabbit polyclonal anti-acetylated Histone H3 (acetyl K9 + K14 + K18 + K23 + K27) (abcam, ab47915, 1:1000). Immunohistochemistry (IHC) or immunofluorescence of FFPE-fixed tumors was performed using established procedures^16^ using anti-Ki67 (clone SP6 Abcam, ab16667, 1:350), anti-E-cadherin (Santa Cruz, sc-7870, 1:75), mouse monoclonal anti-BrdU antibody (MoBU-1 clone, ThermoFisher, B35128, 1:50) and anti-H1.0 (3H9, 1:100) antibodies. To detect H1.0 in colon and hair follicle stem cells, sections from 15 to 20-week-old mice carrying a Lgr5^tm1(cre/ERT2)Cle^ (Lgr5-EGFP-IRES-creERT2)^28^ allele were stained with anti-H1.0 and anti-GFP (Abcam ab6673, 1:1000) antibodies. For TUNEL staining, Promega kit G7130 and G7360 was used in accordance with manufacturer’s instructions. Slides were imaged on Zeiss Axio Scope.Z1 scanner and combined images of the whole section were generated using Zeiss Zen 2 (Blue Edition) V2.1 software. For BrdU staining, paraffin sections were rehydrated and boiled in Tris-EDTA antigen retrieval buffer (10 mM Tris Base, 1 mM EDTA Solution, 0.05% Tween 20, pH 9.0) for 15 min and allowed to cool for 1 hr. Slides were incubated in 2 N HCl for 15 min at 37 °C followed by 0.1 M Sodium Borate pH 8.8 for 20 min. Antibody staining was performed as described above. Tissue sections were imaged using a Zeiss Confocal 710 Upright microscope.

Flow cytometry analysis was performed using anti-CD44-FITC (IM7) (eBioscience, 11-0441-81, 0.5 mg per test) and anti-CD24-VioBlue (clone: 32D12) (Miltenyi biotech, 130-099-150, 1:11). HCC1569 cells were treated with DMSO or Quisinostat for 24 h. Cells were counted and resuspended at a concentration of 1 × 10^6^ cells per 100 µl in sorting buffer (PBS + 5 mM Hepes + 1% BSA + 100 U ml^−^^1^, 2 mM EDTA). Cells were stained with antibodies for 30 min on ice, washed three times with sorting buffer and analyzed using LSR Fortessa (BD bioscience). FACSDiva v8.0 and FlowJo v10 software were used to acquire and analyze data, respectively. The gating strategy is described in Supplementary Fig. 8.

For western blot analysis, anti-acetylated lysine antibody (Cell Signaling technology, 9441S,1:1000), anti-phospho-Smad2 (Ser465/467) (Cell Signaling technology, 3108, 1:500), anti-Smad2/3 (BD, 610843, 1:1000), anti-E-cadherin (clone 36, BD, 610181, 1:1000), anti-Vinculin (clone VIN-11-5, Sigma, V4505, 1:5000) were used. Indicated samples were treated for 24 h with 2 ng ml^−1^ recombinant TGFβ (TGFβ) or 10 µM TGFβ inhibitor SB431542 (TGFβi) to stimulate or inhibit, respectively, TGFβ signaling or 100 nM of Quisinostat.

### Mass spectrometry analysis

To assess kinetics of core histone hyperacetylation, 4 million breast cancer HCC1569 cells treated with 100 nM Quisinostat for 2 or 24 h, or DMSO were analyzed using the Active Motif Mod-Spec service. Histones were acid extracted, derivatized via propionylation, digested with trypsin, newly formed N-termini were propionylated^48^, and then measured in triplicates using the Thermo Scientific TSQ Quantum Ultra mass spectrometer coupled with an UltiMate 3000 Dionex nano-liquid chromatography system. Data were quantified using Skyline v4.2^49^, and represents the percent of each modification within the total pool of that amino acid residue. The identity of detected acetylated residues is indicated in Supplementary Table 4.

To assess H1.0 acetylation status, 4 million breast cancer HCC1569 cells and lung cancer PC9 cells treated with 100 nM Quisinostat or DMSO for 24 h were analyzed by mass spectrometry after limited proteolysis. Cells were lysed in triton extraction buffer (PBS containing 0.5% Triton X-100 and protease inhibitors) at a cell density of 10^7^cells per ml, for 10 min, histones were acid-extracted in 0.2 N HCl at a cell density of 4 × 10^7^ cells per ml at 4 °C overnight and dialyzed with Slide-A-Lyzer™ MINI Dialysis Device, 2 K MWCO, 0.1 ml (Thermo Scientific™, cat. number: 69580) in 50 mM HEPES Buffer pH7.5

Limited proteolysis: 1 mg aliquots of acid-extracted proteins from each sample were carbamidomethylated then digested in 50 mM HEPES at pH 7.5 with both 10 ng and 100 ng trypsin (modified sequencing grade, Promega) for 1, 4 and 16 h at 37 °C. Digestion was halted by acidifying (pH 2) with trifluoroacetic acid (TFA) and refrigeration. Undigested protein was removed from the digests using 1 mm^3^ 3 M Empore C18 SPE discs (aka “stage tips”) eluting at 45% acetonitrile (ACN)/0.1% formic acid (FA). Extracted tryptic peptides were then dried in a vacuum centrifuge.

For mass spectrometry, samples dissolved in 0.1% TFA were loaded on an Ultimate 3000 nanoRSLC HPLC equipped with a 2 mm × 0.3 mm Acclaim Pepmap C18 trap column at 15 µl min^−1^ of 0.1% TFA before elution at 0.25 µl min^−1^ via a 50 cm × 75 μm EasySpray C18 column coupled to an Orbitrap Fusion Lumos (all columns and instruments from Thermo Scientific). Binary solvent gradients of 8–25%B in 50′; 2–40%B in 35′; 40–60%B in 5′; followed by cleaning and re-equilibration, were run over 120 mins (A = 2% ACN, 0.1% FA; B = 80% ACN, 0.1% FA). The Orbitrap was operated in “Data Dependent Acquisition” mode with a survey scan at a resolution of 120 k from m z^−^^1^ 300–1500, followed by MS/MS in “TopS” mode. Dynamic exclusion was set to 30 s with max. charge set to 1*e*^6^ ions in 10 ms. MS/MS spectra were acquired in the ion trap using HCD fragmentation. AGC was set to 2*e*^3^ ions in 300 ms for all available parallelizable time.

For analysis, raw files were processed using Maxquant (v1.6.0.13)^50^ to search the uniprot *Homo* *sapiens* reference proteome with visualization in Perseus (v1.4.0.2)^51^. A decoy database of reversed sequences was used to filter false positives, with an FDR of 1% for both peptides and proteins. Detected peptides for histones proteins, including H1.0, H1.4, H2, H3, H4 were selected from the modificationSpecificPeptides.txt table and the number of unmodified or acetylated peptides quantified, for both untreated (0.1% DMSO) and treated (100 nM Quisinostat) cells.

### Chromatin immunoprecipitation (ChIP)

Ten million HCC1569 cells were fixed with 1% formaldehyde for 10 min at RT, treated with 125 mM glycine for 5 min at RT and washed three times with PBS. Pellets were resuspended in 1.8 ml IP buffer (100 mM Tris at pH 8.6, 100 nM NaCl, 0.25% SDS, 2.5% Triton X-100, and 5 mM EDTA) and incubated for 20 min on ice. Chromatin was sheared to 200–500 bp with 5 cycles of 0.5 s ON/OFF using the Bioruptor pico sonicator (Diagenode), quantified using Bradford assay and 100 mg of sonicated chromatin was used for each immunoprecipitation. Immunoprecipitation was carried out overnight at 4 °C with either H3K27ac (10 µg, Abcam, ab4729), H3K9ac (10 µg, Cell Signaling, 9649S) or IgG (10 µg, Abcam, ab46540). Immune complexes were recovered by adding 30 μl of magnetics bead (Dynabeads Protein G, Life Technologies) and incubated for 3 h at 4 °C with agitation. Beads were washed three times in 0.5 ml of low salt buffer (20 mM Tris at pH 8.1, 150 mM NaCl, 2 mM EDTA, 1% Triton X-100, and 0.1% SDS), and once in 0.5 ml of high salt buffer (20 mM Tris at pH 8.1, 1% Triton X-100, 500 mM NaCl, and 2 mM EDTA, 0.1% SDS). Chromatin-antibody complexes were eluted using elution buffer (120 μl of 1% SDS, 0.1 M NaHCO3) and incubated at 65 °C overnight. DNA was purified using the Qiagen PCR Purification Kit, resuspended in 200 μl of water and 2 μl were analyzed by qPCR.

### siRNA-mediated knockdown

HDAC-targeting siRNAs were aliquoted into 384-well plates at a concentration of 375 nM in HBSS buffer using an Xrd-384 reagent dispenser in (FuidX, 2.5 µl per well). 2.5 µl of Opti-MEM containing 0.5 µl of Lipofectamine RNAiMAX were then dispensed into the wells and the siRNA-lipid complex allowed to form by incubating at room temperature for 15 min. Reverse transfection was performed by seeding 1,300 HCC1569 cells in 45 µl of full culture media in each siRNA-containing well. Plates were incubated at 37 °C 5% CO_2_ for 72 h before fixation and staining with an anti-H1.0 antibody.

### Cell proliferation assay

Cells were harvested by trypsinization, plated at 2 × 10^4^ (HCC1569) or 8 × 10^3^ (TDF) cells per well on 24-well tissue culture plates (Corning, 3526) and incubated at 37 °C and 5% CO_2_ overnight. Cells were then treated with the indicated compound concentration. For experiments in which cell growth was assessed after removal of Quisinostat, 500 cells were plated to allow tracking of individual cells over time. Photomicrographs were taken every 3, 6 or 24 h using an IncuCyte Zoom live cell imager (Essen Biosciences, Sartorius) and % confluence of the cultures measured using the IncuCyte software (v2019b) (Essen Biosciences, Sartorius) over the indicated time in culture. Results were normalized to the % of confluence at the first time point. The effect of H1.0 knockdown is dependent on the dose of Quisinostat used and the phenotype induced by the drug: it rescues proliferation when cytostatic doses of Quisinostat are used; it does not rescue cell survival when high, cytotoxic doses are used. For long-term experiments, medium was changed every 3 days. The number of replicates for each experiment is indicated in figure legends. For cell death analysis IncuCyte® Cytotox Green Reagent (Essen Biosciences, Sartorius) was added to cell culture media to a final concentration of 250 nM following manufacturer’s instructions. For experiments involving treatment with EGFRi (500 nM Osimetinib mesylate, Insight Biotechnology Limited, HY-15772A) or anti-HER2 antibody (20 µg ml^−1^ Trastuzamab, Generon, HY-P9907), cells were re-dosed with drug in fresh media twice a week. For PC9 cells containing an inducible H1.0-targeting shRNA (PC9 + H1.0 KD), doxycycline (1 µg ml^−1^) was added to the media for the entire course of the experiment. After 14 days, all drug-containing media was removed, cells were washed two times with PBS and treatment was switched to 10 nM Quisinostat, drug-free media (0.1% DMSO) or sustained EFGRi/anti-HER2 treatment as indicated in Fig. 6a. DMSO- and drug-containing media was changed three times a week until the end of the experiment.

### In vitro self-renewal assay

HCC1569 cells and cells derived from lung (LXFL1674), mammary (MAXF MX1) and pancreatic (PAXF1997) PDXs were seeded at low density in ultra-low adherent (ULA) round bottom 96-well plates (Corning, 7007). Limiting-dilution assays were performed to ensure that single clonogenic cells were seeded in each well (cell densities giving rise to only 10–30% populated wells in DMSO-treated cells). Performing clonogenic assays in 96-well plates and scoring the percentage of populated wells avoids artefacts due to cell aggregation that may confound the quantification. 72 h after seeding, Quisinostat (12.5, 25 or 50 nM), TGFβi (10 or 30 μM), Quisinostat (12.5 nM) + Doxycycline (1 μg ml^−^^1^) or 0.1% DMSO were added to 96-well plates (one 96-well plate per treatment for each sample). DMSO- and drug-containing media were changed three times a week. After 24 days of treatment, 96-well plates were scanned using IncuCyte S3 (Essen Biosciences, Sartorius) and presence or absence of spheres scored for each cell line and treatment condition. The effect of H1.0 knockdown is dependent on the dose of Quisinostat used and the phenotype induced by the drug: it rescues self-renewal when cytostatic doses of Quisinostat are used; it does not rescue cell survival when high, cytotoxic doses are used.

### Tumor maintenance assays

HCC1569 breast cancer cells were injected in the mammary fat pad (two injections, right and left site per mouse) of eight 8–10-week-old NSG female mice to generate orthotopic xenografts (1 × 10^6^ cells in 50 μl of 75% matrigel-reduced growth factors, Scientific Laboratory Supplies, 356231). Lung (LXFL1674), mammary (MAXFMX1) and pancreas (PAXF1997) PDXs (Oncotest, Charles River) were propagated by transplanting 5 × 10^5^ cells into both flanks of six 8–10-week-old NSG mice. When tumors reached 4–5 mm in diameter, mice were randomly split into 2 groups for daily treatment with either Quisinostat (4 mg kg^−^^1^) or vehicle (20% hydroxypropyl-β-cyclodextrin) by intraperitoneal injections. Tumor growth was monitored twice weekly by bilateral caliper measurements and tumor volume calculated. When assessing the effect of H1.0 KD, three groups of 8 mice were injected with the same number of HCC1569 cells containing uninduced shH1.0. After tumors reached ~5 mm in size, mice were treated with 2 or 4 mg kg^−^^1^ Quisinostat or vehicle for 21 days. Four mice per group were also treated with 2 mg ml^−1^ Doxycycline-1% sucrose in drinking water (changed every 2–3 days) to induce H1.0 KD during Quisinostat treatment. After treatment, tumors were excised and either dissociated for self-renewal assays or fixed and analyzed by immunohistochemistry. Animal studies were subject to ethical review by the Francis Crick Animal Welfare and Ethical Review Body and regulation by the UK Home Office project licence PPL 70/8167.

### In vivo self-renewal assays

For dissociation, Quisinostat- or vehicle-treated tumors were chopped with a blade into 2–3 mm pieces, introduced in a gentleMACS c-tubes (Miltenyi Biotec, 130-093-237) containing 4 volumes of 0.5× liberase (Roche, 5401127001) in warm RPMI media, and blended on a gentleMACS dissociator with program h_tumor_0.1. After a 30 min incubation at 37 °C in agitation, program h_tumor_0.2 was run, 25 μl of DNAseI (New England Biolabs, 2000 U ml^−1^, M0303L) were added and samples were incubated at 37 °C for an additional 30 min in agitation. After running program h_tumor_0.3, samples were filtered using a 70 μm strainer (Sigma, CLS431751-50EA) and washed three times with RPMI media. Cells from four treated tumors per condition were pulled together to assess the average frequency of self-renewing cells across tumors. Graded numbers of alive tumor cells were resuspended in 50 μl of 75% matrigel (Scientific Laboratory Supplies, 356231) and injected in the mammary fat pad of 8–10-week-old NSG female mice. No doxycycline was administered to recipient mice, even when assessing tumors in which H1.0 had been knocked down. Tumor appearance was monitored for 4 months. Frequency of self-renewing cells in treated tumors was estimated by limiting-dilution analysis using the ELDA webtool http://bioinf.wehi.edu.au/software/elda/.

For analysis of colon and hair follicle stem cell function, mice were administered BrdU by intraperitoneal injections (Calbiochem, 203806, 10 mg ml^−^^1^, 10 μl g^−^^1^ body weight) and tissues were harvested 6 h later. For colon analysis, full cross-sections of colonic crypts were bisected at the midline and position of first cell away from the midline was designated 1. Both sides of 10 crypts were quantified per mouse and the frequency at a given position was summed. Animal studies were subject to ethical review by the Francis Crick Animal Welfare and Ethical Review Body and regulation by the UK Home Office project licence PPL 70/8167.

### Bone marrow isolation and analysis

Femurs were obtained from mice immediately after cervical dislocation. Femurs were punctured at both ends and centrifuged to pellet marrow cells in PBS + 10% FBS. Bone marrow cells were incubated with red blood cell lysis buffer (155 mM NH_4_Cl, 12 mM NaHCO_3_, 0.1 mM EDTA) for 5 min at room temperature to remove erythrocytes. Remaining cells were lineage depleted with an EasySepMouse Haematopoietic Progenitor Isolation Kit (Stem Cell Technologies #19856). Lineage depleted cells were stained in PBS + 5% FBS + brilliant stain buffer (BD #563794) with the following antibodies: Lineage cocktail (BV421, Biolegend #133311), CD117 (PEcy7, eBioscience #25117182), Sca1 (APC, eBioscience #17598183), CD34 (FITC, BD #553733), CD135 (PE, BD #553842), CD48 (APCcy7, Biolegend #103432), CD150 (BV650, Biolegend #115931), CD16/32 (APCcy7, Biolegend #101328) and CD127 (BV711, Biolegend #135035). Cells were washed thoroughly and analyzed on a BD LSR Fortessa FACS analyser. The gating strategy is described in Supplementary Fig. 8.

For colony-forming assays, lineage negative cells obtained from the bone marrow were counted and 10,000 cells were plated in methylcellulose semi-solid medium (MethocultGF M3434, Stem Cell Technologies). Cells were allowed to grow for 7 days and counted using an upright microscope to score colony type and number as per the manufacturers’ instructions.

### Analysis of tumor relapse

To examine the effect of Quisinostat on EGFRi-surviving human NSCLC cells in vivo, H1975 lung cancer cells were injected intradermally in both flanks of four 8–10-week-old NSG female (1.5 ×10^6^ cells in 100 μl of PBS). When tumors were 8–12 mm in diameter, mice were treated daily with EGFRi (Osimetinib mesylate, Insight Biotechnology Limited, 25 mg kg^−1^ diluted in 0.5% hydroxy propyl methyl cellulose) via oral gavage for 16 days followed by daily IP injections with 4 mg kg^−^^1^ Quisinostat or vehicle (20% hydroxypropyl-β-cyclodextrin). Animals were allocated to either vehicle or Quisinostat making sure that tumors in the two conditions were comparable before starting the new treatment. Tumor growth was monitored twice weekly by bilateral caliper measurements and tumor volume calculated. A second experiment performed using eight NSG females showed results very similar to the first one.

Tetracycline-inducible EGFR^L858R^ mice (allele name: Tg(tet-O-EGFR*L858R)56Hev) were obtained from the Mouse Repository of the National Cancer Institute. The R26tTA (Gt(ROSA)26Sor^tm1(tTa)Roos^) and Trp53^fl/fl^ (Trp53^tm1Brn^) mice were obtained from the Jackson laboratory. Where relevant, mice were backcrossed to a C57Bl/6J background using the MaxBax protocol (Charles River, Harlow UK). Mice were crossed to generate two cohorts of mice: (i) Rosa26tTa^LSL^ tet(O)EGFR^L858R^ and (ii) Rosa26tTa^LSL^tet(O)EGFR^L858R^ Trp53^flox/flox^. After weaning, the mice were genotyped (Transnetyx, Memphis, USA), and placed in groups of one to five animals in IVCs, with a 12-h daylight cycle. Cre-expressing adenovirus (Viral Vector Core, University of Iowa, USA) was used to induce lung specific recombination and expression of human EGFR^L858R^, and simultaneously inactivate Trp53, depending on the genotype. Viral particles were delivered via intratracheal intubation (single dose, 2.5 × 10^7^ virus particles in 50 µl of DMEM medium per single mouse). Mice were between 3 and 7 months old at the time of viral induction and were induced on the same day, using the same suspension of viral particles. All animal experiments were subject to ethical review by the Francis Crick Animal Welfare and Ethical Review Body and regulation by the UK Home Office project licence P8AA77917. Mice were weighed once per week and injected with 25 mg kg^−^^1^ EGFRi (Erlotinib, Selleckchem, Cat. No. S1023, dissolved in 0.3% (hydroxypropyl)methyl cellulose) via intraperitoneal injection, once a day, five days a week, weekends without therapy. After one month of EGFRi treatment, mice were switched to 4 mg kg^−1^ Quisinostat (Generon, HY-15433), or vehicle (20% hydroxypropyl-β-cyclodextrin, Sigma, H107) with the same schedule as for EGFRi.

For tumor tracking through time and volumetric measurements, mice’s thorax were scanned using Bruker, Skyscan 1176 under isoflurane anesthesia. Scans were performed once a month. Images were processed using RespGate v0.3c for respiratory gating, NRecon v1.6.10.4 for *z* stack image reconstruction. For viewing and image tumor volume calculation a combination of CT-Analyser v1.10.11.0+ and DataViewer v1.5.2.4 was used. Tumor volumes were plotted as percentage of the volume measured at the start of EGFRi treatment.

### RNA sequencing and quantitative RT-PCR

For RNA-seq analysis, samples were treated with DMSO or 100 nM Quisinostat and harvested after 30 min, 1 h, 3 h and 10 h of treatment. RNA extraction was carried out using RNeasy Plus Mini Kit (Qiagen) following the manufacturer’s instructions. RNA libraries were prepared using KAPA mRNA Hyper Prep Kit (Roche), assessed on a DNA 1000 BioAnalyser 2100 chip (Agilent) to ensure good quality, and sequenced on an Illumina HiSeq 4000 sequencer, generating ~25 million 76-bp strand-specific single-end reads per sample. Adapter trimming was performed with cutadapt^52^ (v1.9.1)^52^ with parameters “--minimum-length=25 --quality-cutoff=20 -a AGATCGGAAGAGC”. The RSEM package^53^ (v1.2.31) in conjunction with the STAR alignment algorithm^54^ (v2.5.2a) was used for the mapping and subsequent gene-level counting of the sequenced reads with respect to hg19 RefSeq genes downloaded from the UCSC Table Browser^55^ on 7th June 2017. The parameters used were “--star-output-genome-bam --forward-prob 0”. Differential expression analysis was performed with the DESeq2 package^56^ (v1.14.1) within the R programming environment (v3.3.2). An adjusted *p*-value of FDR ≤ 0.01 was used as the significance threshold for the identification of differentially expressed genes (DEGs). Genes with a maximum transcript per million (TPM) value lower than 1 across all the samples in the experiment were discarded. To identify HDACi-responsive, H1.0-dependent genes, genes showing differential expression between Quisinostat-treated and DMSO-treated cells at each time point were first selected. Among this group of DEGs, genes differentially expressed between uninduced Quisinostat-treated cells and dox-induced Quisinostat-treated cells were then selected, ensuring that gene expression changes were opposite to those induced by Quisinostat (upregulated in Quisinostat vs DMSO and downregulated in Quisinostat _dox vs Quisinostat, and vice versa).

For qRT-PCR, cDNA was generated using High Capacity cDNA Reverse Transcription Kits (Life Technologies) and gene expression levels were analyzed on a CFX96 real-time PCR detection system (Bio-rad) using SsoAdvanced™ Universal SYBR® Green Supermix (Bio-rad), primers listed in Supplementary Table 3 and CFX manager 3.0 software. Cyclophilin A (*PPIA*) was used as reference housekeeping gene. Expression levels of H1 variants upon Quisinostat treatment are indicated in Supplementary Table 5.

### Gene set enrichment analysis

HDACi-responsive, H1.0-dependent genes were analyzed using the “compute overlaps” function of the GSEA (v7.0) Molecular Signature Database (MSigDB) (http://software.broadinstitute.org/gsea/msigdb/index.jsp), focusing on the hallmark gene sets. Enrichment of gene signatures was considered biologically significant if *p*-value was equal or lower than 10^−6^. Upregulated and downregulated genes at any time point were analyzed independently.

### Statistical analysis

Unless otherwise stated in figure legends, data are presented either as individual samples or as mean ± standard error of the mean (s.e.m.) of multiple replicates, with *N* indicated in the figure legend. Appropriate statistical tests were performed to assess the significance of the differences between samples. The statistical test used for each comparison, whether they were one‐ or two‐sided, whether adjustment for multiple corrections was performed and the *p*‐value are indicated in the corresponding figure legends. Source data for all graphs can be found in the Source data file.

### Reporting summary

Further information on experimental design is available in the Nature Research Reporting Summary linked to this paper.

## Supplementary information

Supplementary Information

Description of Additional Supplementary Information

Supplementary Data 1

Supplementary Data 2

Supplementary Data 3

Reporting summary

## Data Availability

The accompanying RNA-seq data set is available through GEO: GSE119369. Source data for all graphs and blots are available in the Source Data File. Results of the compound screen are in Supplementary Dataset 1. All other data are available on request from the authors.

## Source data

Source Data File
