# Supplementary material for: Selective inhibition of cancer cell self-renewal through a Quisinostat-histone H1.0 axis
Source: Nat Commun. 2020 Apr 14;11:1792. doi: 10.1038/s41467-020-15615-z (PMC7156485; doi:10.1038/s41467-020-15615-z)
Supplement: Supplementary file 1 — Supplementary Information [file 41467_2020_15615_MOESM1_ESM.pdf]

SUPPLEMENTARY INFORMATION

Selective inhibition of cancer cell self-renewal through a Quisinostat-histone H1.0 axis

Morales Torres et al.

Contents:

Supplementary Figure 1-8
Supplementary Table 1-5
Supplementary References

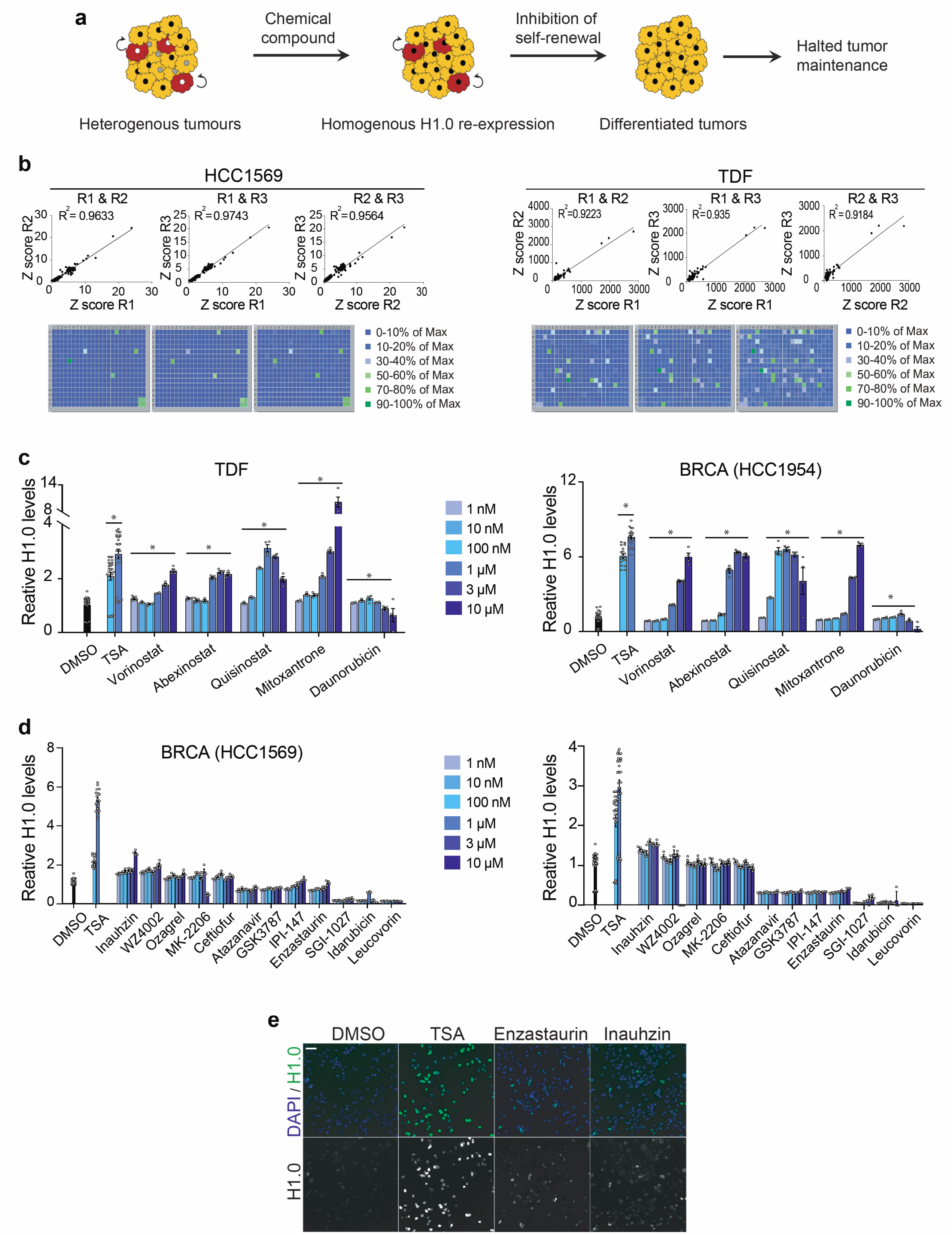

Supplementary Figure 1 High-throughput compound screening identifies HDAC inhibitors as modulators of H1.0 levels

a. Schematic representation of the rationale behind the high-throughput compound screen. Red and yellow shapes represent self-renewing and differentiated tumor cells, respectively. White, grey and black circles represent nuclei containing low, medium and high H1.0 levels, respectively.

b. Consistency among replicates in the screen. Correlation between the Z scores of positive hits between the indicated replicates (R1, R2 and R3) (above) and heatmap of H1.0 signal intensity for a representative plate showing highly consistent replicates (below). Results for both cell lines used in the screen are shown.

c. Validation experiments showing a dose-dependent increase in H1.0 levels upon treatment with the indicated compounds in the indicated cell lines. Values represent mean ± s.e.m from four biological replicates for each condition, except DMSO, TDF (N = 60); DMSO, HCC1954 (N = 56); 100nM and 1μM TSA, TDF (N = 30); 100nM TSA and 1μM TSA, HCC1954 (N = 18). One asterisk indicates p-value < 0.0001 (one-way ANOVA) for each drug titration compared to DMSO.

d-e. Analysis of other primary hits. Quantitative immunofluorescence microscopy measuring H1.0 levels upon treatment with the indicated compounds. Weak activity was observed for some compounds. For other molecules, activity was not confirmed. Values represent mean ± s.e.m from four biological replicates for each condition, except HCC1569, DMSO (N = 48), HCC1569, 100nM TSA (N = 24); HCC1569, 1μM TSA (N = 18); TDF, DMSO (N = 60); , TDF, 100nM TSA and 1μM TSA (N = 30). Representative images showing moderate increase in H1.0 levels upon treatment with the indicated compounds. Similar results were obtained in four independent experiments (e). Nuclei were counterstained with DAPI. Scale bar: 50 μm.
Source data are provided as a Source Data file.

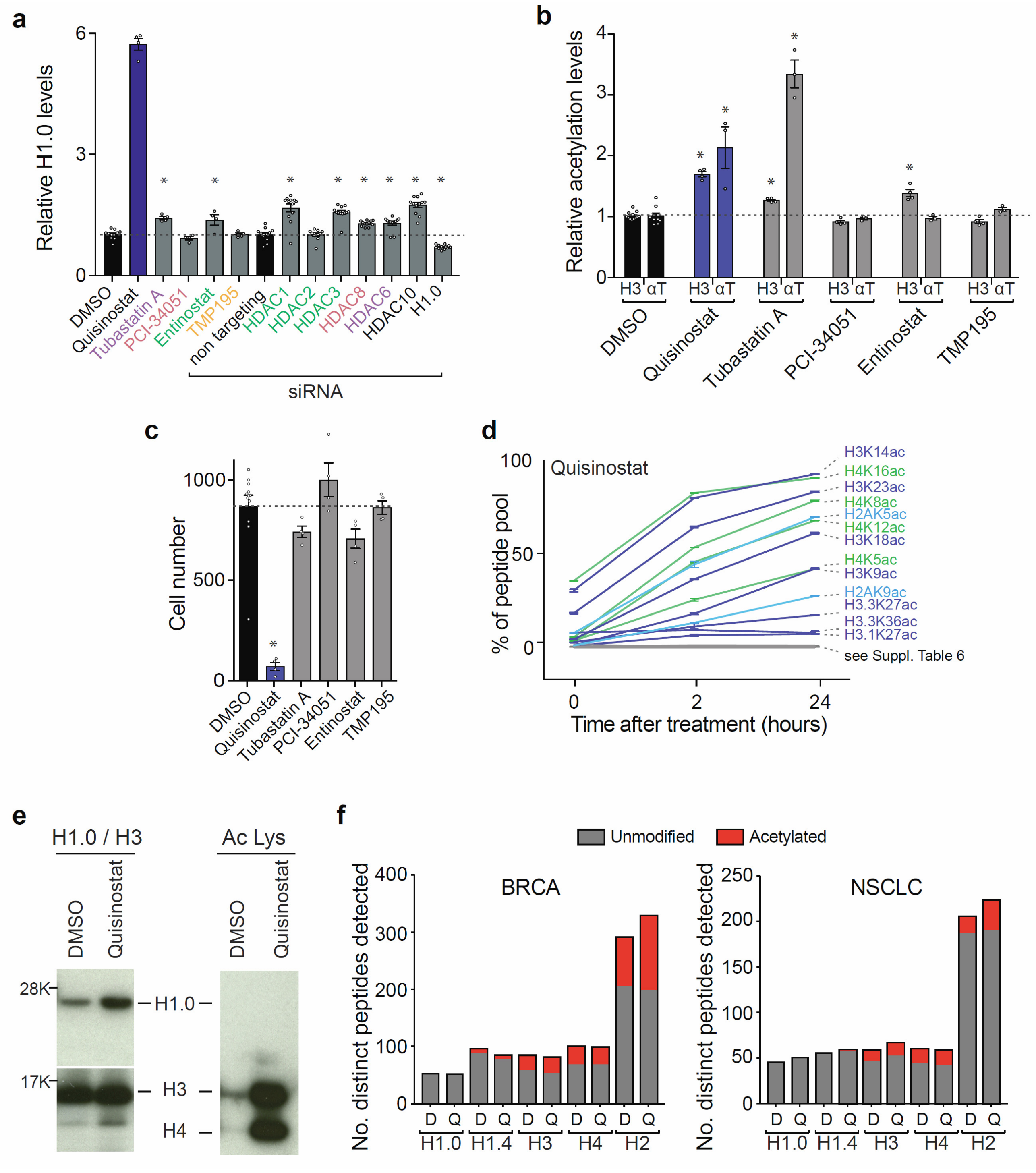

Supplementary Figure 2 Inhibition of multiple HDACs is required for robust H1.0 upregulation

a. Immunofluorescence microscopy quantifying H1.0 levels 24 h after treatment of HCC1569 cells with the indicated HDACi (10 µM) or 72h after knockdown of the indicated HDACs. Levels are compared to those induced by the broad-spectrum inhibitor Quisinostat. Colors indicate which HDACs are inhibited by the specific inhibitors. Lower doses did not show any effect except for Quisinostat. Values represent mean ± s.e.m from four or twelve biological replicates for HDACi- and siRNA-treated samples, respectively, except DMSO (N =11). One asterisk indicates p-value < 0.001 (one-tailed Student's t-test) compared to

DMSO (inhibitors) or non-targeting siRNAs (HDACi-targeting siRNAs). Exact p-values are in the Source Data file.

b. Immunofluorescence microscopy quantifying the levels of acetylated H3 (H3, detected modifications: K9ac + K14ac + K18ac + K23ac + K27ac) and acetylated alpha-tubulin (αT) 24 h after treatment of HCC1569 cells with the indicated HDACi. Levels are compared to those induced by the broad-spectrum inhibitor Quisinostat. Values represent mean ± s.e.m from 4 (AcH3) or 3 (Ac-α-tub) biological replicates. For DMSO, N = 12 and 10 biological replicates, respectively. One asterisk indicates p-value < 0.01 (one-tailed Student's t-test) compared to DMSO. Exact p-values are in the Source Data file.

c. Quantification of cell number 24 h after treatment of HCC1569 cells with the indicated HDACi. Levels are compared to those induced by the broad-spectrum inhibitor Quisinostat. Values represent mean ± s.e.m from 4 biological replicates. For DMSO, N = 12 biological replicates. One asterisk indicates p-value < 0.001 (one-tailed Student's t-test) compared to DMSO. Exact p-values are in the Source Data file. All compounds affected cell number at later time points confirming their activity.

d. Quantification of the relative abundance of the indicated acetylated residues by mass spectrometry at the indicated times after 100 nM Quisinostat treatment. Residues are color-coded based on histone protein. Grey lines indicate modifications with maximal value < 2, listed in Supplementary Table 4.

e. Analysis of acid-extracted histones from HCC1569 cells treated with 100 nM Quisinostat for 24 h or DMSO by Western blot. Membranes were probed with anti-H1.0 or anti-H3 (left) and anti-acetyl Lysine (Ac Lys, right) antibodies. No band corresponding to acetylated H1.0 was detected. Similar results were obtained in two independent experiments and were replicated with non-small cell lung cancer cells PC9.

f. Analysis of acid-extracted histones from HCC1569 cells (left, BRCA: breast invasive carcinoma) or PC9 cells (NSCLC: non-small cell lung cancer) treated with 100 nM Quisinostat (Q) for 24 h or DMSO (D) by mass spectrometry. The number of distinct unmodified or acetylated peptides detected in each condition is plotted. While numerous acetylated peptides were detected for core histones and for the H1.4 variant of the linker histone, none were detected for H1.0. Note that the abundance of each peptide is not taken into account in this quantification, unlike the analysis shown in panel d. This explains why DMSO- and Quisinostat-treated samples do not show major differences.

Source data are provided as a Source Data file.

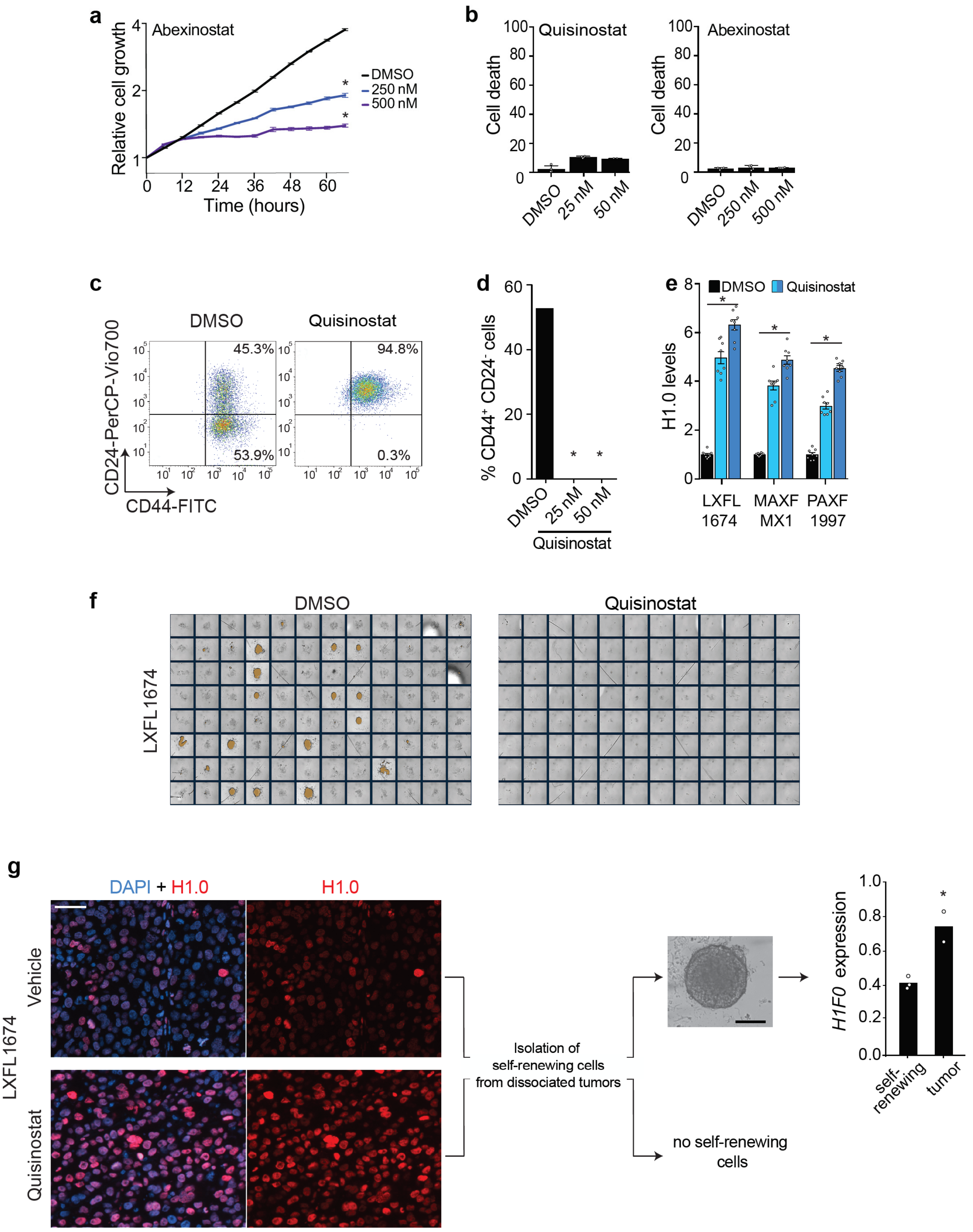

Supplementary Figure 3 Quisinostat treatment inhibits cancer cell self-renewal

a-b. IncuCyte proliferation assay measuring growth kinetics (a) and cell death (% of surface area occupied by dead, Cytotox Green positive cells) at 66h (b) of HCC1569 cells treated with the indicated HDACi. Values

represent mean ± s.e.m from four biological replicates. One asterisk indicates p-value < 0.001 (one-tailed Student's t-test) calculated at the final time point of each treatment compared to DMSO (a). Exact p-values are in the Source Data file. Similar results were obtained with TDF cells.

c-d. Flow cytometric analysis of the indicated surface antigens of HCC1569 cells upon a 5 day treatment with Quisinostat. Self-renewing breast cancer cells are characterized by a $CD44^{+}/CD24^{-}$ immunophenotype (c). Quantification of $CD44^{+}/CD24^{-}$ cells upon treatment of HCC1569 cells with the indicated doses of the Quisinostat for 5 days (d). One asterisk indicates p < 0.0001 (two-way contingency table analysis and two-tailed Fisher's exact test).

e. Quantitative immunofluorescence microscopy measuring H1.0 levels in the indicated PDX-derived cells upon treatment with 100 nM (light blue) and 1 µM (dark blue) Quisinostat. Values represent mean ± s.e.m from eight biological replicates. One asterisk indicates p-value < 0.0001 (one-way ANOVA) comparing Quisinostat- and corresponding DMSO-treated cells.

f. Representative example of clonogenic assays using cells isolated from the LXFL 1674 PDX model of lung cancer. The yellow area indicates detected colonies.

g. Analysis of Quisinostat-induced effects on the LXFL 1674 PDX model of lung cancer. Tumors treated with vehicle or 4 mg Kg^{-1} Quisinostat for 15 days were either stained to detect H1.0 or dissociated to isolate self-renewing cells as spheroids by growth in low adherent conditions. No self-renewing cells could be isolated from Quisinostat-treated tumors. Self-renewing cells were then compared with the whole tumor population by qRT-PCR (right), showing particularly low levels of H1.0, as previously reported [1]. Note that H1.0 levels are highly heterogeneous in the tumor bulk of vehicle-treated tumors (left), which results in moderate differences when averaging values across the population by qRT-PCR. Scale bar: 50 µm (immunofluorescence images), 500 µm (phase contrast). qRT-PCR values represent mean from three technical replicates. One asterisk indicates p-value < 0.05 (one-tailed Student's t-test). Exact p-values are in the Source Data file.

Source data are provided as a Source Data file.

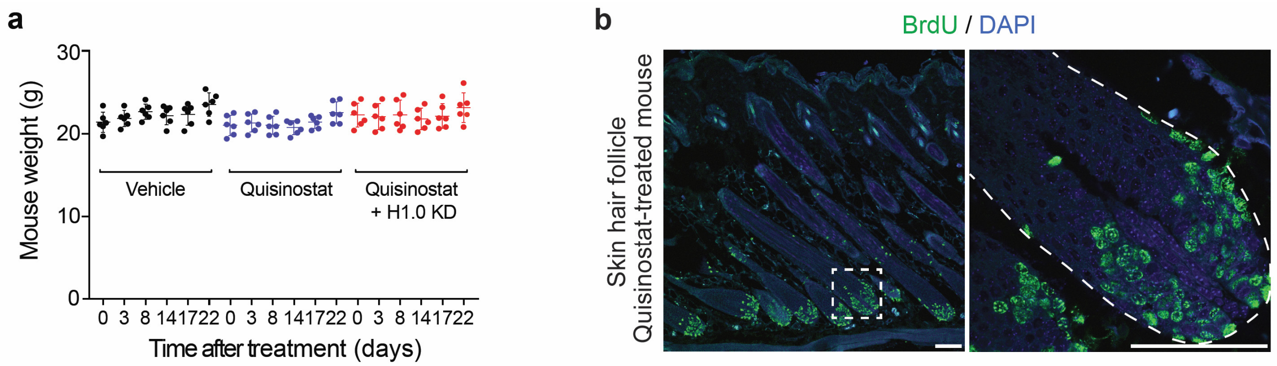

Supplementary Figure 4 Normal tissue homeostasis in Quisinostat-treated mice

a. Assessment of mouse well-being based on the absence of weight loss during Quisinostat treatment. Each dot represents a mouse treated with either vehicle of Quisinostat. The condition Quisinostat + H1.0 KD relates to experiments shown in Fig. 4. Values are mean ± s.e.m from six mice per condition.

b. Immunodetection of dividing cells (BrdU$^+$, green) in the hair follicle bulge in Quisinostat-treated mice. Cell nuclei are marked by DAPI (blue). The area in the white square is shown at higher magnification on the right. Dotted line in the right panel indicates hair follicle. Scale bars: 100 µm. Three out of three mice treated with Quisinostat showed BrdU labelling in the hair follicle bulge.

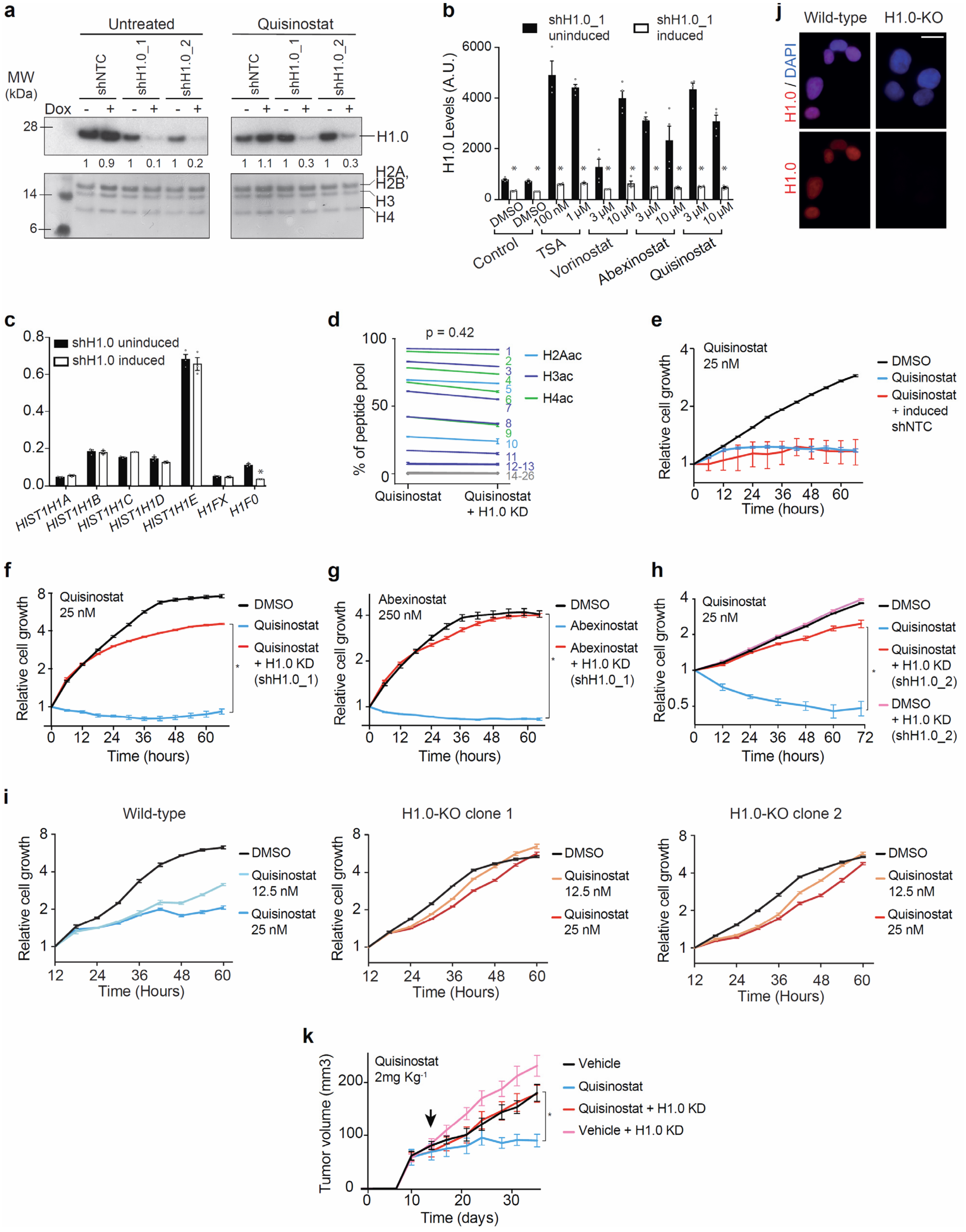

Supplementary Figure 5 H1.0 knockdown abrogates the anti-tumor effect of multiple HDACi

a. Western blot analysis of the indicated samples (HCC1569 cells) using an anti-H1.0 antibody. Core histones are visualized by Ponceau S staining. Numbers indicate the relative levels of H1.0 in each induced

sample (+ Dox) compared to the corresponding uninduced sample (values are normalized to the levels of H2A and H2B in each lane). H1.0 levels are slightly reduced in the untreated, uninduced condition (- Dox) due to leaky expression of shRNA. Acid-extracted histones are loaded. The small difference between the Quisinostat-treated shNTC samples is likely due to minor technical variability in chromatin extraction efficiency. Quisinostat dose: 100 nM for 8h. Dox: doxycycline. shNTC: non-targeting control shRNA. MW: molecular weight.

b. Quantification of H1.0 levels by quantitative immunofluorescence microscopy in HCC1569 cells treated with the indicated HDACi for 24h and expressing (white) or not expressing (black) an H1.0-targeting shH1.0 (shH1.0_1). Values are mean ± s.e.m from four biological replicates except shH1.0 uninduced, 100 nM TSA (N = 3). One asterisk indicates p-value < 0.05 (one-tailed Student's t-test) compared to the corresponding uninduced condition. Exact p-values are in the Source Data file.

c. Quantification of the expression levels of the indicated genes encoding H1 variants upon H1.0 knock-down. No compensatory upregulation is observed. qRT-PCR values represent mean from three technical replicates. One asterisk indicates p-value < 0.05 (Unpaired two-tailed Student's t-test). Exact p-values are in the Source Data file. Similarly, H1.0 upregulation by Quisinostat does not lead to compensatory downregulation of other H1 variants (Supplementary Table 5).

d. Quantification of the relative abundance of modified core histone residues by mass spectrometry 24 h after Quisinostat treatment. HCC1569 expressing (Quisinostat + H1.0 KD) or not expressing (Quisinostat) shH1.0_1 are compared. Residues are color-coded based on histone protein. Grey lines indicate modifications with maximal value < 2. The identity of the residues corresponding to the numbers is indicated in Supplementary Table 4. The significance of the overall difference between the two conditions is indicated (two-tailed paired Student's t-test).

e. IncuCyte proliferation assay measuring growth kinetics of HCC1569 cells treated with Quisinostat. Cells contain an inducible non-targeting shRNA construct, which is either not expressed (Quisinostat) or expressed (Quisinostat + induced shNTC). The corresponding H1.0 KD graph is in Figure 4a. Values represent mean ± s.e.m from four biological replicates, except DMSO (N=12).

f-h. IncuCyte proliferation assay measuring growth kinetics of TDF (f, g) and HCC1569 (h) cells treated with the indicated HDACi. Cells contain two distinct inducible H1.0-targeting shRNA (shH1.0_1 in f and g, shH1.0_2 in h), which are either not expressed (Quisinostat/Abexinostat) or expressed (Quisinostat/Abexinostat + H1.0 KD). The graph for HCC1569 containing shH1.0_1 is in Figure 4a. shH1.0_1 and shH1.0_2 have been extensively characterized and shown to elicit similar functional and molecular effects both *in vitro* and *in vivo,* in a specific manner [1]. A control in which H1.0 is knocked-down in the absence of Quisinostat treatment is shown in h. The differences in Quisinostat effect between panel h and e (blue lines) is due to slight differences in activity between two Quisinostat batches. Values represent mean ± s.e.m from four biological replicates per condition (f-h), except DMSO in g (N = 8); DMSO and DMSO +H1.0 KD (shH1.0_2) in h (N = 3). One asterisk indicates p-value < 0.001 (one-tailed Student's t-test) calculated at the last time point. Exact p-values are in the Source Data file.

i. IncuCyte proliferation assay measuring growth kinetics of wild-type or H1.0 knocked-out (H1.0-KO) TDF cells treated with the indicated doses of Quisinostat or DMSO as a control. Two distinct H1.0-KO clones are shown. Results are normalized to the % of confluence after 12 hours of treatment. Values represent mean ± s.e.m from four biological replicates (see source data).

j. Immunofluorescence microscopy of wild-type and H1.0-KO (Clone 1) TDF cells using and anti-H1.0 antibody. Similar results were obtained with H1.0-KO clone 2. Scale bar: 10 μm.

k. Tumor maintenance assay. Growth kinetics of HCC1569-induced tumors treated with 2mg kg^{-1} Quisinostat or vehicle. Injected cells contain an inducible H1.0-targeting shRNA (shH1.0_1) which is either not expressed (Quisinostat) or expressed to prevent H1.0 upregulation (Quisinostat + H1.0 KD). Values represent mean ± s.e.m from eight tumors. One asterisk indicates p-value < 0.001 (one-tailed Student's t-test) calculated at the final time point. Exact p-values are in the Source Data file. The arrow indicates when Quisinostat treatment was started. Results obtained administering 4mg kg^{-1} Quisinostat are shown in Fig. 4d. A control in which H1.0 is knockdown in the absence of Quisinostat treatment is shown. As previously reported, preventing spontaneous re-expression of H1.0 in the tumor bulk results into more aggressive tumors that contain an increased fraction of self-renewing cells [1]. This effect is specific to H1.0 and control shRNAs have no effects [1].
Source data are provided as a Source Data file.

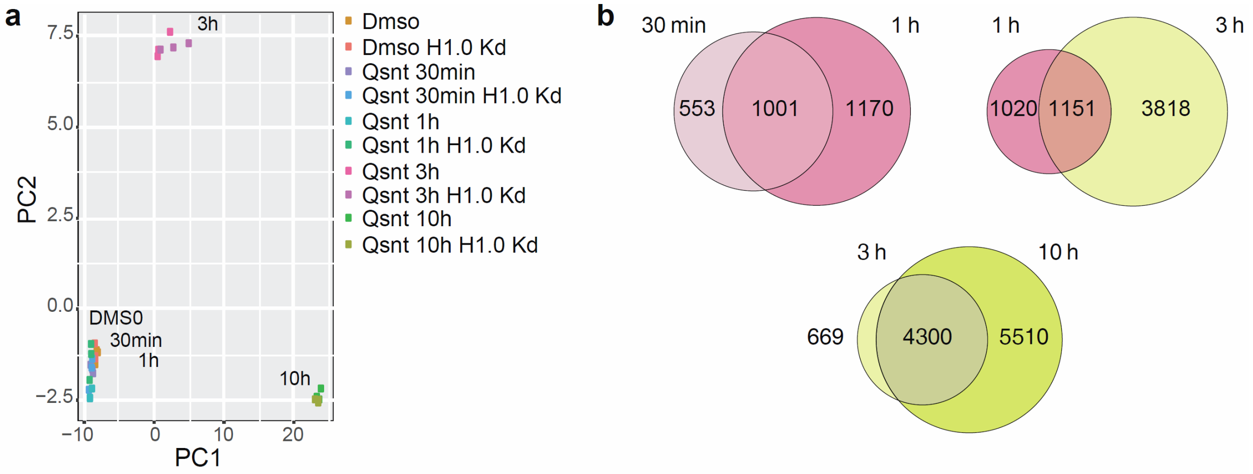

c

	30 min		1 h		3 h		10 h	
	Gene Set name	FDR q-value	Gene Set name	FDR q-value	Gene Set name	FDR q-value	Gene Set name	FDR q-value
Upregulated			Interferon-γ response	5.13E-08	TNF-α via NFkB	2.81E-12	Cholesterol homeostasis	6.79E-08
			TNF-α via NFkB	1.20E-06	Interferon-γ response	5.60E-08	Apoptosis	3.71E-07
					Apoptosis	2.22E-07	TNF-α via NFkB	1.33E-06
							Androgen response	4.39E-06
Downregulated	**TGF-β signaling**	2.07E-11	MYC targets	1.19E-12	Apical junction	1.91E-08	E2F targets	2.53E-19
	EMT	2.07E-11	EMT	1.05E-10	EMT	1.69E-07	MYC targets	2.53E-19
	**Hypoxia**	2.07E-11	TNF-α via NFkB	1.05E-10	UV Response DN	1.69E-07	G2M checkpoint	5.58E-17
	**TNF-α via NFkB**	2.07E-11	**MTORC1 signaling**	1.53E-08			Mitotic spindle	4.55E-08
	Apical junction	9.60E-07	TGF-β signaling	1.80E-07			Apical junction	3.55E-07
	IL2-STAT5 signaling	9.60E-07	Apoptosis	1.80E-07			Coagulation	7.07E-07
			G2M checkpoint	1.04E-06			Estrogen response	1.76E-06
			Mitotic spindle	1.04E-06			Hypoxia	1.76E-06
			Androgen response	7.58E-06			P53 pathway	1.76E-06
			Glycolysis	7.58E-06			Androgen response	4.30E-06
			Hypoxia	7.58E-06				
			**PI3K-AKT-TOR**	7.61E-06				

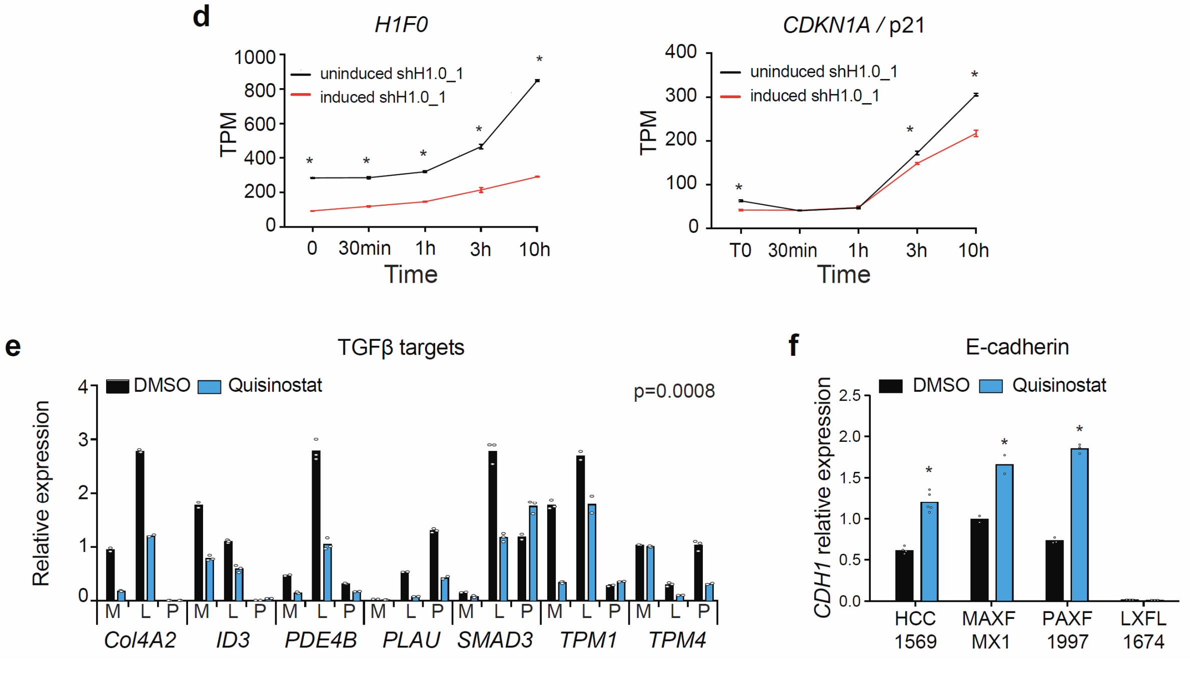

Supplementary Figure 6 Identification of Quisinostat-responsive, H1.0-dependent genes

a. Principal component analysis of the characterized samples.
b.Quantification of Quisinostat-responsive genes at the indicated time points after Quisinostat treatment.
c. Gene signatures enriched among Quisinostat-responsive, H1.0-dependent genes at the indicated time points. Gene signatures are from the GSEA hallmark sets. Signaling pathways previously linked to EMT are marked in bold.
d. Expression levels of the indicated genes in cells expressing or not expressing shH1.0_1 at the indicated times after Quisinostat treatment. Values represent mean ± s.e.m from three biological replicates. One asterisk indicates p-value < 0.01 (one-tailed Student's t-test). Exact p-values are in the Source Data file.
e-**f**. qRT-PCR measuring expression levels of TGFβ targets (e) and *CDH1/*e-cadherin (f) in cells derived from the indicated PDXs upon Quisinostat treatment. p-value is indicated on the figure (paired two-tailed Student's t-test). M, L and P indicate MAXFMX1, LXFL1674 and PAXF1997, respectively. Note the widespread downregulation of TGFβ targets, despite slight PDX-specific differences, indicating overall inactivation of the pathway (e). The lung PDX showed minimal levels of basal E-cadherin expression likely reflecting a non-epithelial origin. The absence of upregulation upon treatment is thus not surprising. Values represent mean from three (LXFL1674, PAXF1997), two (MAXFMX1) and four (HCC1569) technical replicates. One asterisk indicates p-value < 0.05 (one-tailed Student's t-test). Exact p-values are in the Source Data file (f).
Source data are provided as a Source Data file.

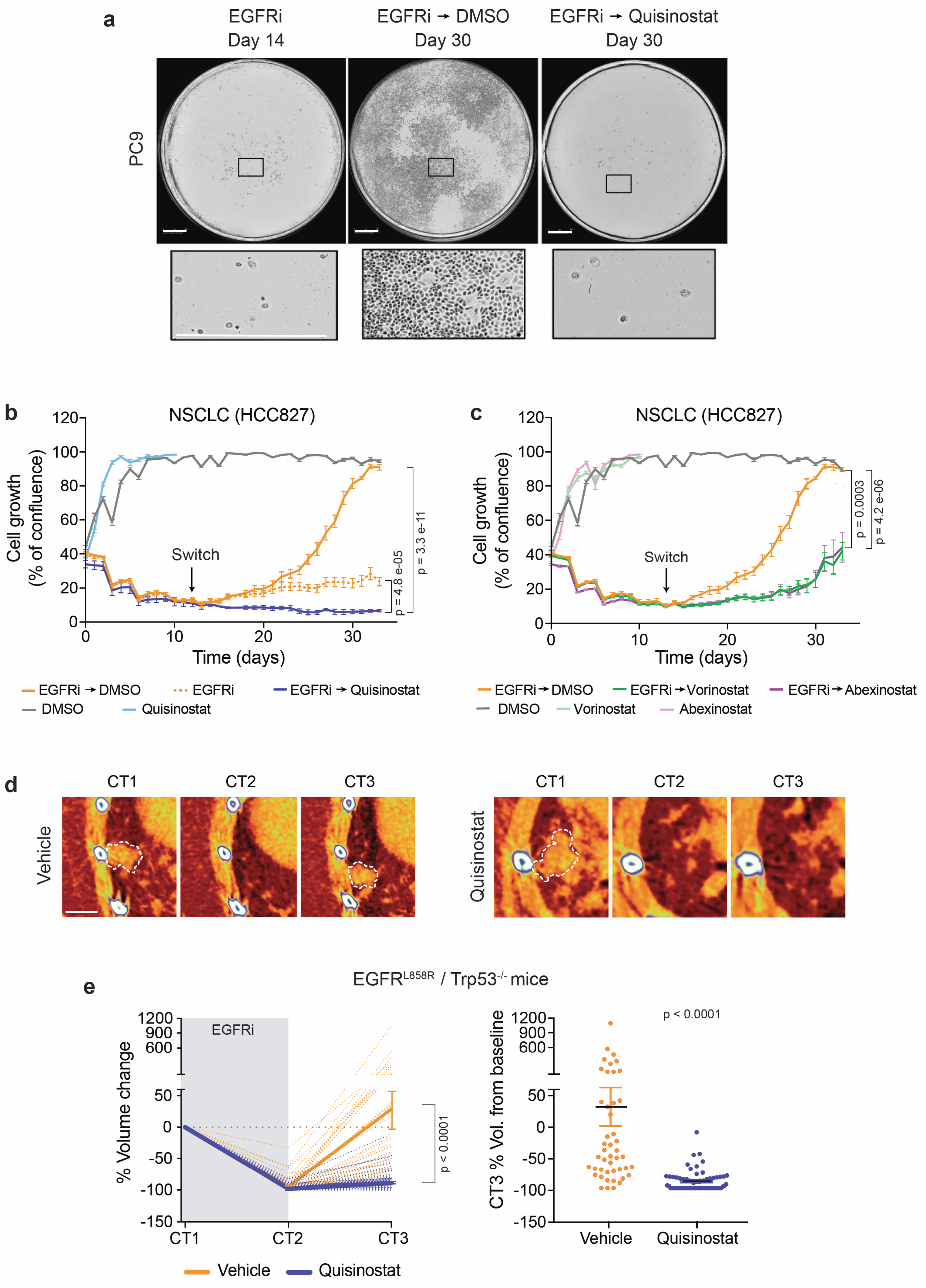

Supplementary Figure 7 Weaker effect of Vorinostat and Abexinostat on EGFRi-surviving cells.

a. Representative images of PC9 cells treated with the indicated compounds at the indicated time after EGFRi treatment. Panels at the bottom are higher magnification images of the cells in the black rectangles. Scale bar: 1 mm

b, c. IncuCyte proliferation assay measuring growth kinetics of non-small cell lung cancer (NSCLC) HCC827 cells treated with the indicated compounds, as explained in Fig. 6a. Compound concentrations were: EGFRi (osimertinib): 500 nM, Quisinostat: 10 nM, Vorinostat: 300 nM, Abexinostat: 100 nM. The line steps correspond to time points of media change. Values represent mean ± s.e.m from 5 biological replicates. p-value: one-tailed Student's t-test. Similar results were obtained in three other experiments. Note that subthreshold concentrations of HDACi, which do not affect cycling cells untreated with EGFRi, are effective in the sequential treatment.

d. Representative CT scans of $EGFR^{L858L}$ mice treated with vehicle or 4mg kg^{-1} Quisinostat after EGFRi treatment (25 mg kg^{-1} Erlotinib) at the indicated CT scans, indicating a relapsed tumor after EGFRi therapy (left) and a tumor that remained undetectable after sequential therapy (right). Detected lung tumors are indicated by a dotted white line. Unlabelled orange areas within the lungs are bronchi and blood vessels. Similar results were obtained in several other mice quantified in e. Scale bar: 2 mm.

e. Response to sequential EGFRi - Quisinostat therapy in $EGFR^{L858L}$ / $Trp53^{-/-}$mice, expressed as percentage of tumor volume change relative to the start of treatment over time (left) and at endpoint (right). Tumors were treated with EGFRi (grey area) and subsequently with Quisinostat or vehicle. CT scans were performed at 1 month intervals. Dotted lines indicate individual tumors and solid lines the average values for each condition ± s.e.m (left). Each dot is an individual tumor, with the black line indicating the mean value (right). N = 46 for vehicle and 66 for Quisinostat. p-value: two tailed Mann-Whitney test.

Source data are provided as a Source Data file.

Gating Strategy Supplementary Fig 3c.

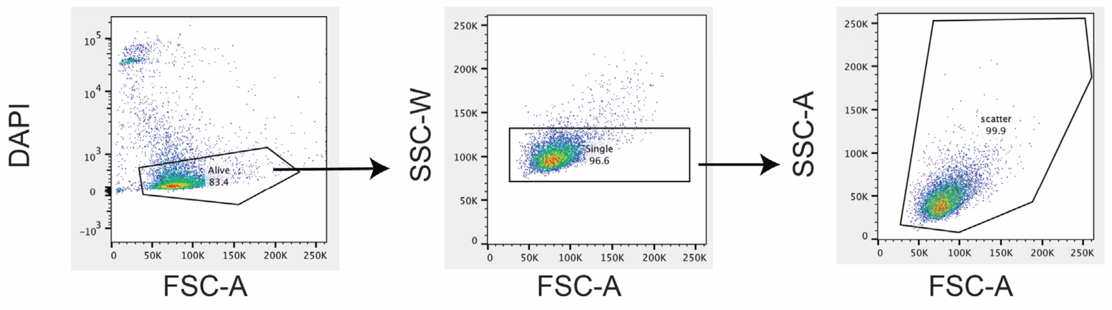

Gating Strategy Fig 2h.

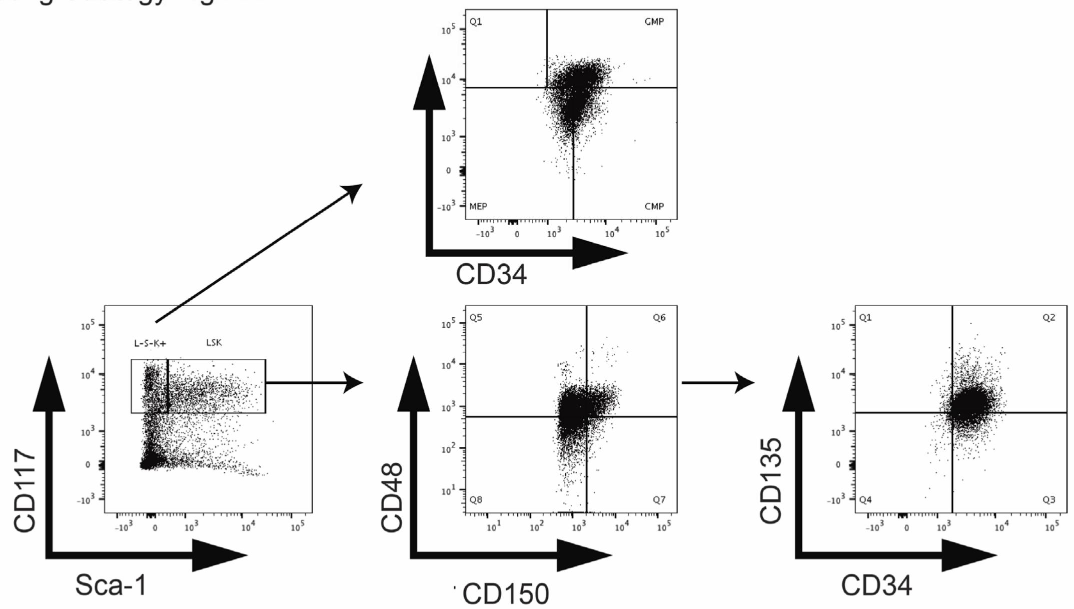

Supplementary Figure 8 Gating strategy for FACS experiments reported in the indicated panels.

Supplementary Table 1. Cell line growth conditions

Cell name	Number in Figure 1d	Medium	Fetal Bovine Serum (FBS)	Non-Essential Amino Acid (NEAA)	L-glutamine	Penicillin	Streptomycin
HCC1569		Roswell Park Memorial Institute medium (RPMI)	10%	-	2 mM	100 U ml^{-1}	100 µg ml-1
HCC1954		RPMI	10%	-	2 mM	100 U ml^{-1}	100 µg ml-1
M059K	2	DMEM+ Ham's F12 (1:1)	10%	1%	2 mM	100 U ml^{-1}	100 µg ml-1
LN-229 (CRL2611)	3	DMEM	5%	-	2 mM	100 U ml^{-1}	100 µg ml-1
U-87 MG	1	DMEM	10%	1%	2 mM	100 U ml^{-1}	100 µg ml-1
C32	4	DMEM	10%	-	2 mM	100 U ml^{-1}	100 µg ml-1
CFPAC-1	6	RPMI	10%	-	2 mM	100 U ml^{-1}	100 µg ml-1
PANC-1	5	DMEM	10%	-	2 mM	100 U ml^{-1}	100 µg ml-1
HCC4006	12	RPMI	10%	-	2 mM	100 U ml^{-1}	100 µg ml-1
PC9	13	RPMI	10%	-	2 mM	100 U ml^{-1}	100 µg ml-1
HCC827	14	RPMI	10%	-	2 mM	100 U ml^{-1}	100 µg ml-1
H1975	15	RPMI	10%	-	2 mM	100 U ml^{-1}	100 µg ml-1
786-0	17	RPMI	10%	-	2 mM	100 U ml^{-1}	100 µg ml-1
SK-HEP-1	7	DMEM	10%	-	2 mM	100 U ml^{-1}	100 µg ml-1
HEC-1-A	8	DMEM	10%	-	2 mM	100 U ml^{-1}	100 µg ml-1
HCT116	9	DMEM	10%	-	2 mM	100 U ml^{-1}	100 µg ml-1
SW480	10	DMEM	10%	-	2 mM	100 U ml^{-1}	100 µg ml-1
SK-OV-3	11	DMEM	10%	-	2 mM	100 U ml^{-1}	100 µg ml-1
TDF		MEM	15%	-	2 mM	100 U ml^{-1}	100 µg ml-1
LXFL 1674		RPMI	15%	-	2 mM	100 U ml^{-1}	100 µg ml-1
MAXF MX1		RPMI	15%	-	2 mM	100 U ml^{-1}	100 µg ml-1
PAXF 1997		RPMI	15%	-	2 mM	100 U ml^{-1}	100 µg ml-1
BT474	16	RPMI	10%	-	2 mM	100 U ml^{-1}	100 µg ml-1

All cells were grown at 37 °C in 5% CO_2

Supplementary Table 2. Patient-derived-xenograft information

	LXFL 1674	PAXF 1997	MAXFTN MX1
Cancer type	NSCLC (large cell subtype)	pancreatic cancer	breast cancer (triple negative)
Patient histology	large cell carcinoma	adeno carcinoma	not available
Gender	female	male	female
Patient Age at Surgery	45	79	29
Ethnicity/ Strain	Caucasian	Caucasian	African (predicted)
Primary/ Metastasis/ Recurrent	Primary	Primary	not known
Site of origin	Lung	Pancreas	not known
Patient Tumor Differentiation	poor	moderate	poor
Stage at Implantation	not available	pT3, pN0 (0/7), pMx. L1. V1.	not available
Pre-Implantation Chemo/Radiotherapy	not known	no	not known
PDX Stroma content	15.0 %	6.0 %	4.0 %
PDX Vascularization	intermediate	low	high
PDX Differentiation	undifferentiated	poor	poor

Supplementary Table 3. Sequence of oligonucleotides used in the study

qRT-PCR Primers

Gene	Fw primer (5'-3')	Rv primer (5'-3')
TGFB1	GCAAGTGGACATCAACGGG	GCTGAAGCAATAGTTGGTGTCC
PLAU	CGACTCCAAAGGCAGCAATG	TGCTGCCCTCCGAATTTCTT
SMAD3	GGTCAAGAGCCTGGTCAAGA	TTGAAGGCGAACTCACACAG
TPM1	GCAAATGTGCCGAGCTTGAA	CTGCGAGTACTTCTCAGCCT
COL4A2	GGATGGCTATCAAGGGCCTG	CTGGCACCTTTTGCTAGGGA
H1F0	CTGGCTGCCACGCCCAAGAA	CGGCCCTCTTGGCACTGGAC
PDE4B	CTTCTCTCTGTTTGCCTGCC	ACCACTGCTCCTTTCTACCC
PPIA(Cyclophilin)	GTCAACCCCACCGTGTTCTT	CTGCTGTCTTTGGGACCTTGT
CHD1	AAGGGGTCTGTCATGGAAGG	GGTGTTCACATCATCGTCCG
ID3	GGCCCCCACCTTCCCATCC	GCCAGCACCTGCGTTCTGGAG
TPM4	CAAGGAGAATGCCATCGACC	GTTTCTTCTGGAGGTGCGTC
HIST1H1A	CTCCTCTAAGGAGCGTGGTG	GAGGACGCCTTCTTGTTGAG
HIST1H1B	GTCAAAAAGGTGGCGAAGAG	CTTGGCCTTTGCAGCTTTAG
HIST1H1C	ACACCGAAGAAAGCGAAGAA	GCTTGACAACCTTGGGCTTA
HIST1H1D	GGAGACTGCTCCACTTGCTC	GCCTTCTTCGCCTTTTTCTT
HIST1H1E	TTCCGGCTCGAATTGCTCTC	CTTCACGGGAGTCTTCTCGG
H1FX	GTGGTTCGACCAGCAGAATG	GAGCTTGAAGGAACCGTTGG

ChIP primers

Gene	Fw primer (5'-3')	Rv primer (5'-3')
H1F0 Promoter	CAGCAACAAACTCCCACTCC	CCCACAACCCCGCTTTTATT
Chr4p15.31	ATATGCTCAACCCTTGCCCT	GGCTACTGTGGAATGGAACG

Supplementary Table 4. Identity of acetylated residues detected by Mass Spectrometry.

1	H3: K14AC
2	H4: K16AC
3	H3: K23AC
4	H4: K8AC
5	H2A: K5AC
6	H4: K12AC
7	H3: K18AC
8	H3: K9AC
9	H4: K5AC
10	H2A: K9AC
11	H3.3: K27AC
12	H3.3: K36AC
13	H3.1: K27AC
14	H1.4: K25AC
15	H2A: K36AC
16	H2A1: K13AC
17	H2A1: K15AC
18	H2A3: K13AC
19	H2A3: K15AC
20	H3R2UN: K4AC
21	H3: K56AC
22	H3: K64AC
23	H3: K79AC
24	H3: K122AC
25	H3.1: K36AC
26	H4: K20AC

The table refers to data shown in Supplementary Figures 2d and 5c. Redidues are color-coded as in the graphs.

Supplementary Table 5. Expression levels of H1 variants upon Quisinostat treatment as assessed by qRT-PCR.

Target	Treatement	Time point	Normalized expression	SEM
HIST1H1A	DMSO	T0	0.043538301	0.00351304
	100 nM Quisinostat	30'	0.03527802	0.00217314
		1h	0.046546713	0.00333133
		3h	0.03206811	0.00149532
		10h	0.035123292	0.00249189
HIST1H1B	DMSO	T0	0.181333331	0.01786094
	100 nM Quisinostat	30'	0.155519655	0.0102348
		1h	0.174697525	0.02156669
		3h	0.192084803	0.01630932
		10h	0.334474604	0.04087964
HIST1H1C	DMSO	T0	0.149996015	0.00711995
	100 nM Quisinostat	30'	0.13743016	0.01661595
		1h	0.168229184	0.01287314
		3h	0.10900273	0.00830653
		10h	0.150304146	0.01723009
HIST1H1D	DMSO	T0	0.142582782	0.01400582
	100 nM Quisinostat	30'	0.139329312	0.0118347
		1h	0.168765091	0.01209025
		3h	0.166666962	0.01120904
		10h	0.132638927	0.01652753
HIST1H1E	DMSO	T0	0.678958461	0.05209018
	100 nM Quisinostat	30'	0.649061834	0.05391579
		1h	0.826347453	0.08233317
		3h	0.731220724	0.04848942
		10h	0.884581554	0.07616038
H1FX	DMSO	T0	0.046984489	0.00536411
	100 nM Quisinostat	30'	0.033667668	0.00324882
		1h	0.049743994	0.00505748
		3h	0.044653186	0.00460869
		10h	0.041380546	0.00284407
H1F0	DMSO	T0	0.108574575	0.01099298
	100 nM Quisinostat	30'	0.106455585	0.00669354
		1h	0.121904273	0.02169799
		3h	0.167957786	0.01382098
		10h	0.260148785	0.04436453

Values are normalized to the houskeeping gene *PPIA* and are mean from three technical replicates.

SUPPLEMENTARY REFERENCES

1. Torres, CM *et al.* The linker histone H1.0 generates epigenetic and functional intratumor heterogeneity. *Science* **353**, (2016).
