# Supplementary material for: Selective inhibition of cancer cell self-renewal through a Quisinostat-histone H1.0 axis
Source: Nat Commun. 2020 Apr 14;11:1792. doi: 10.1038/s41467-020-15615-z (PMC7156485; doi:10.1038/s41467-020-15615-z)
Supplement: Supplementary file 2 — Description of Additional Supplementary Information [file 41467_2020_15615_MOESM2_ESM.pdf]

**DESCRIPTION OF ADDITIONAL SUPPLEMENTARY ITEMS**

File Name: Supplementary Data 1
Description: Compound screen

File Name: Supplementary Data 2
Description: Primary and validated hits

File Name: Supplementary Data 3
Description: HDAC inhibitors targets
